# Semi-Synthesis and Biological Evaluation of 25(*R)*-26-Acetoxy-3*β*,5*α*-Dihydroxycholest-6-One

**DOI:** 10.3390/md21030191

**Published:** 2023-03-20

**Authors:** Mireguli Maimaitiming, Ling Lv, Xuetao Zhang, Shuli Xia, Xin Li, Pingyuan Wang, Zhiqing Liu, Chang-Yun Wang

**Affiliations:** 1 Institute of Evolution & Marine Biodiversity, School of Medicine and Pharmacy, College of Food Science and Engineering, Ocean University of China, Qingdao 266003, China; 2 Laboratory for Marine Drugs and Bioproducts, Qingdao National Laboratory for Marine Science and Technology, Qingdao 266237, China

**Keywords:** steroid, semi-synthesis, anti-RSV activity, anti-cancer activity, cytotoxicity, apoptosis

## Abstract

Previously, we identified a series of steroids (**1**–**6**) that showed potent anti-virus activities against respiratory syncytial virus (RSV), with IC_50_ values ranging from 3.23 to 0.19 µM. In this work, we first semi-synthesized and characterized the single isomer of **5**, 25(*R*)-26-acetoxy-3*β*,5*α*-dihydroxycholest-6-one, named as (25*R*)-**5**, in seven steps from a commercially available compound diosgenin (**7**), with a total yield of 2.8%. Unfortunately, compound (25*R*)-**5** and the intermediates only showed slight inhibitions against RSV replication at the concentration of 10 µM, but they possessed potent cytotoxicity activities against human bladder cancer 5637 (HTB-9) and hepatic cancer HepG2, with IC_50_ values ranging from 3.0 to 15.5 µM without any impression of normal liver cell proliferation at 20 µM. Among them, the target compound (25*R*)-**5** possessed cytotoxicity activities against 5637 (HTB-9) and HepG2 with IC_50_ values of 4.8 µM and 15.5 µM, respectively. Further studies indicated that compound (25*R*)-**5** inhibited cancer cell proliferation through inducing early and late-stage apoptosis. Collectively, we have semi-synthesized, characterized and biologically evaluated the 25*R*-isomer of compound **5**; the biological results suggested that compound (25*R*)-**5** could be a good lead for further anti-cancer studies, especially for anti-human liver cancer.

## 1. Introduction

Steroids, containing a basic 17 carbon atom-formed perhydrocyclopentanophenanthrene skeleton, represent a polycyclic compound superfamily including sterol, bile acids, sex hormones, molting hormones of insects, adrenal cortical hormones and other physiologically active substances [1]. Steroids widely exist in plants, animals, microorganisms and other living organisms and possess significant and distinct roles in the construction of cell membranes, cell stability and growth, proliferation, cell function and other cell biological processes [1,2,3]. Therefore, numerous steroid compounds play essential roles in the treatment of various human diseases including cancer, inflammation, infection, metabolic diseases, cardiovascular diseases, heart diseases, neurological disorders and other human diseases [4,5,6,7,8,9,10,11]. Especially, the tetracyclic ring steroid plays a significant role in drug discovery [12,13]. Due to the wide application in human diseases’ therapy and drug discovery programs, the identification of new bioactive steroids has attracted lots of attention from the pharmaceutical industry and academia.

**Figure 1:**
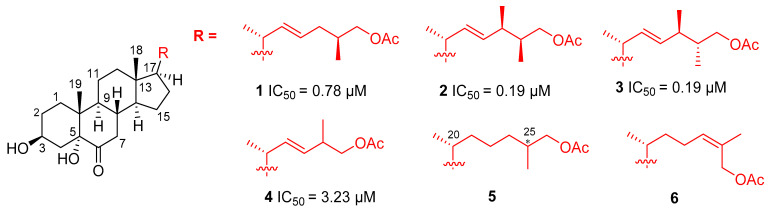
Chemical structures and anti-RSV activities of steroid compounds **1**–**6**.

In recent years, one of our research interests has been to discover novel bioactive steroids with anti-virus, anti-inflammatory, anti-oxidant and anti-cancer activities from marine organisms as chemical probes to influence biological systems or to act as lead compounds for further drug development [14,15,16,17,18,19,20]. Interestingly, a series of steroids (**1**–**6**, Figure 1) were isolated from the gorgonian *Echinogorgia rebekka* from the South China Sea, which showed potent anti-virus activities against respiratory syncytial virus (RSV), with IC_50_s ranging from 0.19 to 3.23 µM [16]. As previously reported, RSV is a severe infectious disease that causes about 60,000 in-hospital deaths every year in children under the age of 5 [21]. Unfortunately, there are no licensed vaccines for RSV. The only FDA-approved drug, palivizumab, has failed to show sufficient efficacy and is accompanied by serious side-effects [21]. Therefore, novel and effective anti-RSV agents are still an urgent need. Meanwhile, our previously reported steroids (Figure 1) showed potent in vitro anti-RSV activities and were supposed to be an excellent anti-RSV lead for further drug development. However, an insufficient amount of these steroids (**1**–**6**, Figure 1) hampers the further exploration, which is a well-acknowledged challenge in this field [22]. Especially, compound **5**, first discovered from gorgonian *Acalycigorgia inermis* in 2000 by the Shin group, was isolated as inseparable C25-epimeric mixtures [23]. The anti-RSV activity of compound **5** was not studied previously, but it exhibited moderate anti-proliferation activity against the human leukemia cell line K-562, with a LC_50_ of 0.9 µg/mL [16,23]. In order to characterize it and to explore the anti-RSV studies of the single isomer of compound **5**, in this work, we semi-synthesized, characterized and biologically evaluated the 25*R*-isomer of compound **5** for the first time.

## 2. Results and Discussion

### 2.1. Chemistry

**Scheme 1:**
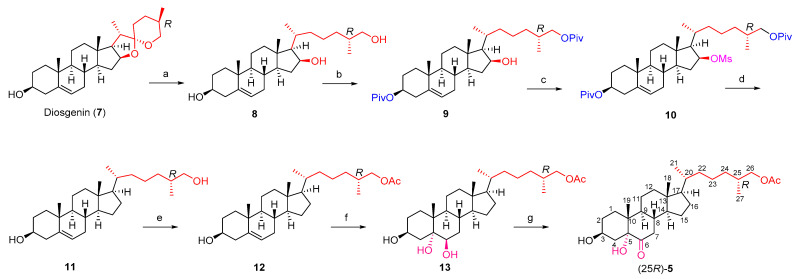
Semi-synthesis of (25*R*)-**5**. Reagents and conditions: (**a**) Zn dust, HCl, EtOH, reflux, 1.5 h, 78%; (**b**) pivaloyl chloride, pyridine, THF, 0-rt, overnight, 49%; (**c**) methanesulfonic anhydride, pyridine, 0-rt, overnight, 99%; (**d**) LiAlH_4_, Et_2_O, reflux, 10 h, 91%; (**e**) acetyl chloride, Et_3_N, DMAP, CH_2_Cl_2_, overnight, 42%; (**f**) (i) magnesium monoperoxyphthalate hexahydrate, acetone, reflux, 30 min; (ii) Bi(OTf)_3_, acetone, rt, 1 h, 41%; (**g**) *N*-bromosuccinimide, acetic acid, acetone, H_2_O, 0-rt, 1 h, 47%.

The synthetic procedure of a single isomer of compound **5**, 25(*R*)-26-acetoxy-3*β*,5*α*-dihydroxycholest-6-one, namely (25*R*)-**5**, is shown in Scheme 1. As outlined in Scheme 1, intermediate **8**, containing three hydroxy groups, was prepared by the reduction of the commercially available nature product diosgenin (**7**) using Zn dust in the mixed solution of HCl and EtOH, with an excellent yield of 78% [24]. Protection of two hydroxy groups of intermediate **8** with the pivaloyl group resulted in compound **9**. Protection of the third hydroxy of intermediate **9** was accomplished under methanesulfonic anhydride in the presence of pyridine as the base, leading to compound **10** [24]. Deprotection and reduction of intermediate **10** with LiAlH_4_ under the condition of reflux produced intermediate **11** with a yield of 91% [24,25]. Protection of the hydroxyl group on the linker terminal of compound **11** by acetyl chloride in the presence of Et_3_N led to intermediate **12** with a moderate yield. Asymmetric dihydroxylation of compound **12** with the magnesium monoperoxyphthalate hexahydrate as the epoxidizing agent, followed by an epoxide opening in the presence of a catalytic amount of Bi(OTf)_3_, provided compound **13** [26,27]. Finally, compound (25*R*)-**5** was obtained by specific mono-oxidation of the key intermediate **13** with *N*-bromosuccinimide in the mixture solution of acetic acid, acetone and water, with a yield of 47% [26].

### 2.2. Characteristic Analysis

The high-resolution electron spray ionization mass (HRMS-ESI) of compound (25*R*)-**5** possessed [M + H]^+^ peak at *m/z* 477.3578 and [M + Na]^+^ peak at *m/z* 499.3401 (Figure S1), corresponding to the reported molecular formula of C_29_H_48_O_5_ of compound **5** [16,23]. Further, the ^1^H and ^13^C NMR spectrometry analysis suggested that our semi-synthesized compound (25*R*)-**5** was a single isomer and the spectrum data (Table 1, Figures S2–S5) were identical to the reported data of compound **5** [23]. As shown in Table 1, the four methyl proton signals in the high-field region were observed at *δ*_H_ 0.91 (d, *J* = 6.7 Hz), 0.90 (d, *J* = 6.5 Hz), 0.79 (s) and 0.64 (s), together with the ^13^C NMR signals at *δ*_C_ 18.6, 16.8, 14.0 and 12.0, respectively. The ^1^H proton signal of the acetoxy group on the linker terminal of the compound was found at *δ*_H_ 2.05 (s), as well as the ^13^C NMR signals at *δ*_C_ 171.4 and 21.0, respectively. The hydroxy group signals of compound **5** were not given in previous reports; we also did not find the two hydroxy group signals in the ^1^H NMR spectrometry. Luckily, when we changed the solvent from CDCl_3_ into DMSO-*d*_6_, the two hydroxy group signals were observed clearly at *δ*_H_ 5.29 (s) and 4.35 (d, *J* = 5.6 Hz) (Figures S4 and S5). Additionally, the chemical shifts (Table 1, Figures S2 and S3) of the steroidal tetracyclic scaffold were all in agreement with reported and our previously identified data. The absolute configuration of compound (25*R*)-**5** was established on the basis of the configuration of previously confirmed compound **12** and detailed ^1^H NMR spectroscopic data, especially the chirality of the C-25 position.

**Figure 2:**
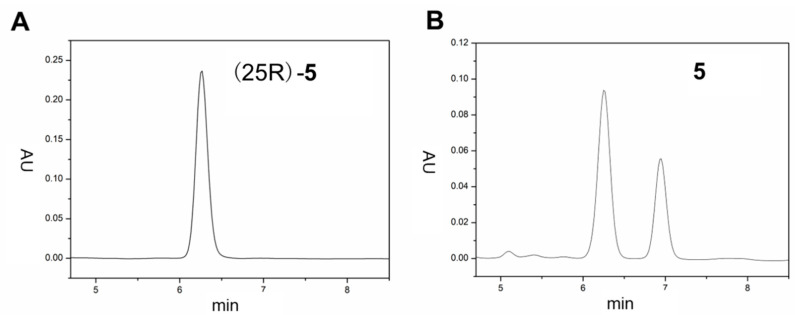
The HPLC chromatograms of compounds (25*R*)-**5** (**A**) and **5** (**B**). HPLC conditions: Waters Xbridge C18 (250 mm × 4.6 mm, 5 µm), flow rate 1.0 mL/min, UV detection at 203 nm and linear gradient from 90% MeOH in water to 100% MeOH in 20 min followed by 30 min of the last-named solvent. The horizontal axis is retention time (min, from 5 to 9 min is shown in these figures) and the axis of ordinate is relative intensity absorbance unit (AU).

As shown in Figure 2 and Figure S6, compounds (25*R*)-**5** and **5** were analyzed by high-performance liquid chromatography (HPLC) to confirm that compound (25*R*)-**5** was one isomer of compound **5**. As expected, compound (25*R*)-**5** only showed one peak, with the retention time of 6.262 min; in contrast, compound **5** possessed two peaks, with the retention times of 6.255 and 6.944 min, respectively.

### 2.3. Biological Studies

With compounds (25*R*)-**5** and the intermediates in hand, their anti-viral activities against RSV were evaluated by a quantitative reverse transcription PCR (RT-qPCR) method in Hep G2 cells, which is a standard method for the anti-viral activity analysis. Unfortunately, all of the tested compounds only showed a slight inhibition on RSV replication at the concentration of 10 µM (Table 2). Although these compounds were not good enough for further anti-RSV studies, they could be seen as good starting points for further structure optimization.

Considering the preliminary anti-proliferation activities against the human leukemia K-562 cell line of compound **5** in previous studies [16,23], we then turned to the exploration of the cytotoxicity activity of compound (25*R*)-**5**. Hence, the semi-synthesized compound (25*R*)-**5**, along with the more drug-like intermediates **8**, **11** and **13** during the synthesis of (25*R*)-**5**, were selected for the biological evaluation of proliferation inhibitory activities against nineteen human cancer and one normal liver cell lines using a CCK-8 assay. First, the anti-proliferation activity of compound (25*R*)-**5** was tested against the human leukemia K-562 cell line, but it only showed a weak inhibition activity at the concentration of 20 µM (Table 3). Meanwhile, all the three intermediates **8**, **11** and **13** did not exhibit any anti-proliferative activities against K-562 cancer cells at 20 µM. As listed in Table 3, similar results were also obtained with nine human cancer cell lines, including HeLa, TE-1, GBC-SD, MCF7, SF126, DU145, CAL-62, HOS and 293T. The intermediate **8** displayed moderate anti-proliferation activity against the human lung adenocarcinoma A549 cell line (53% inhibition rate at 20 µM, Table 3), whereas compound (25*R*)-**5** and intermediates **11** and **13** did not show an inhibition rate over 50%. This result suggested that the hydroxy group at the C-16 position and the double bond of compound **8** had an important role for the anti-proliferation activity against the A549 cell line. Similarly, the same structure–activity relationship (SAR) could also be observed in the human colorectal HCT 116 cancer cell line. Furthermore, all the four compounds exhibited moderate growth inhibitory effects against human gastric MKN-45 and pancreatic PATU8988T cancer cell lines (~50% inhibition rates at 20 µM, Table 3). In Table 3, the four compounds displayed potent activities, with the best inhibition rates over 70% against human melanoma A-375, bladder cancer 5637 (HTB-9) and hepatic cancer HepG2 cell lines. More importantly, all the four compounds had no growth inhibitory effects against the normal human liver L-02 cell line (<10% inhibition rate at 20 µM, Table 3), indicating that these compounds could selectively kill cancer cells. The intermediate **13** displayed less potent cytotoxicity activities than compound (25*R*)-**5** and intermediates **8** and **11**. Therefore, compound (25*R*)-**5** and intermediates **8** and **11** were chosen for further specific cytotoxicity studies.

**Figure 3:**
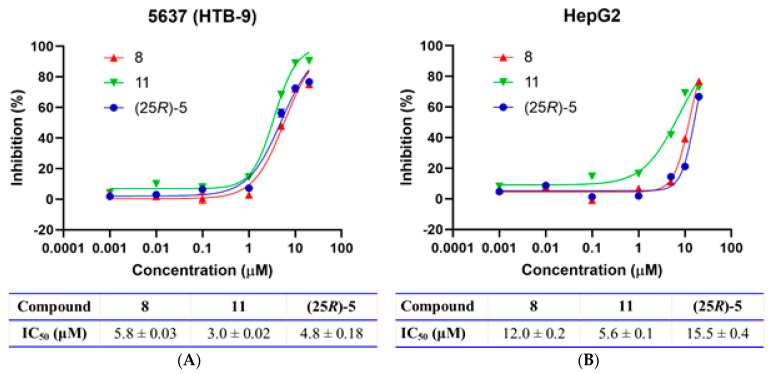
CCK8 assays of 5637 (HTB-9) and HepG2 cells treated with compounds **8**, **11** or (25*R*)-**5** at the indicated concentrations for 48 h. (**A**) Dose-dependent curves of compounds **8**, **11** or (25*R*)-**5** at the indicated concentrations against 5637 (HTB-9) cells. (**B**) Dose-dependent curves of compounds **8**, **11** or (25*R*)-**5** at the indicated concentrations against HepG2 cells. All results are presented as mean ± SEM from triplicate testing in a representative experiment, with similar results observed in at least 3 experiments.

Then, the IC_50_ values of compound (25*R*)-**5** and intermediates **8** and **11** were investigated against 5637 (HTB-9) and HepG2 cancer cell lines, as summarized in Figure 3. As depicted in Figure 3A, all three compounds displayed similar micromolar IC_50_ values (5.8 µM for **8**, 3.0 µM for **11** and 4.8 µM for (25*R*)-**5**, respectively) against 5637 (HTB-9) cancer cells. Interestingly, intermediate **11** proved to be the best compound against HepG2 cancer cells, with an IC_50_ value of 5.6 µM (Figure 3B). Additionally, compound (25*R*)-**5** and intermediate **8** showed very similar anti-proliferation activities against HepG2 cancer cells, with an IC_50_ value of 15.5 µM and 12.0 µM, respectively (Figure 3B). Taken together, compound (25*R*)-**5** represented a promising drug discovery lead towards human liver cancer and is worthy of further discovery.

**Figure 4:**
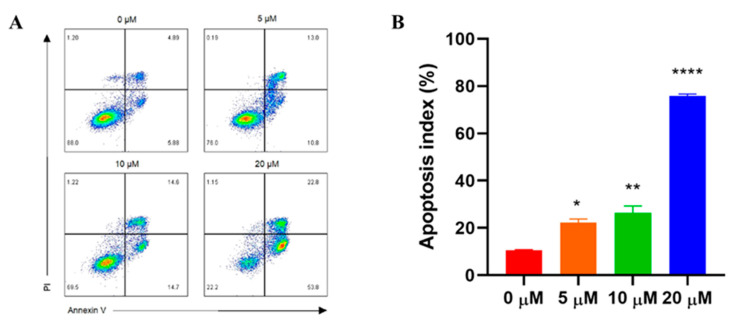
Compound (25R)-5 inhibited the proliferation of and induced apoptosis in 5637 (HTB-9) cells. (**A**) 5637 (HTB) cells were treated with different concentrations of compound (25R)-5 for 48 h, then stained with Annexin-V/PI and analyzed by flow cytometry. (**B**) Quantitative analysis of annexin V-FITC/PI staining after the treatment with (25R)-5 for 48 h, mean ± SEM, *n* = 3; * *p* < 0.05, ** *p* < 0.01, **** *p* < 0.0001; all data in this experiment are expressed as the mean ± SEM of at least three independent experiments.

Next, the apoptosis study of compound (25*R*)-**5** was investigated against 5637 (HTB-9) cells based on a standard flow cytometry technique and assessed by Annexin V-FITC/PI staining. As depicted in Figure 4, the results showed that compound (25*R*)-**5** induced apoptotic cell death in a dose-dependent manner of both early-stage (Annexin VFITC+/PI-) and late-stage (Annexin VFITC+/PI+). Hence, these data strongly suggested that compound (25*R*)-**5** was able to kill cancer cells with the mechanism of inducing cancer cell apoptosis.

## 3. Materials and Methods

### 3.1. Chemistry

All commercially available starting materials and solvents were reagent grade and used without further purification. The diosgenin was purchased from Shanghai Mack-lin Biochemical Co., Ltd., Shanghai, China, with a purity of over 95%. Column chro-matography was carried out on silica gel (200–300 mesh) manufactured by Qingdao Haiyang Chemical Group Co., Ltd. Analytical TLC was performed on silica gel plates and visualized under ultraviolet light (254 nm or 365 nm). 1H and 13C NMR spectra were recorded on a BRUKER AVANCE NEO with 400 MHz for proton (1H NMR) and 100 MHz for carbon (13C NMR). Chemical shifts downfield from TMS were expressed in ppm and the signals are described as d (doublet), dd (doublet of doublet), m (multi-plet), q (quartet), s (singlet) and t (triplet). Coupling constants (J values) were given in Hz. HRMS (ESI) and LC-MS (ESI) were recorded on a SHIMADZU LCMS-IT-TOF mass spectrometer and Thermo TSQ QUANTUM LC-MS spectrometer, respectively. Purity of representative compounds (>95%) was established by 1H NMR and analytical HPLC, which was carried out on a Waters Xbridge C18 (250 mm × 4.6 mm, 5 µm), a flow rate 1.0 mL/min, UV detection at 203 nm and linear gradient from 90% MeOH in water to 100% MeOH in 20 min followed by 30 min of the last-named solvent.

#### 3.1.1. Synthesis of (3*S*,8*S*,9*S*,10*R*,13*S*,14*S*,16*S*,17*R*)-17-((2*R*,6*R*)-7-Hydroxy-6-Methylheptan-2-yl)-10,13-Dimethyl-2,3,4,7,8,9,10,11,12,13,14,15,16,17-Tetradecahydro-1H-Cyclopenta[a]Phenanthrene-3,16-diol (**8**) 

Pre-activated zinc dust (46.1 g, 708 mmol) was added to a solution of diosgenin (2.0 g, 4.8 mmol) in ethanol (100 mL) in a three-necked flask and the mixture solution was heated to reflux. Then, 80 mL of concentrated HCl was added dropwise over 30 min; after the addition of concentrated HCl, the mixture was stirred at reflux for 1 h. After the reaction was completed (detected by TLC), the solution was worked up by the addition of water (100 mL). The excess zinc dust was removed by filtration and washed with CH_2_Cl_2_. The filtrate was extracted with CH_2_Cl_2_ (50 mL × 3) and the combined CH_2_Cl_2_ extracts were washed with brine and dried over anhydrous Na_2_SO_4_. The solution was filtered and condensed by rotary evaporation. The residue was purified by silica gel column chromatography (gradient: 1 to 5% MeOH in CH_2_Cl_2_) to afford compound **8** (1.6 g, 78%, R_f_ = 0.2 in CH_2_Cl_2_: MeOH = 20:1) as a white solid. ^1^H NMR (400 MHz, DMSO-*d*_6_) *δ* 5.25 (d, *J* = 4.6 Hz, 1H), 4.59 (d, *J* = 4.6 Hz, 1H), 4.34 (t, *J* = 5.2 Hz, 1H), 4.27 (d, *J* = 4.9 Hz, 1H), 4.16–4.09 (m, 1H), 3.28–3.20 (m, 2H), 3.17–3.11 (m, 1H), 2.17–2.02 (m, 3H), 1.96–1.18 (m, 16H), 1.09–0.95 (m, 5H), 0.93 (s, 3H), 0.89 (d, *J* = 6.6 Hz, 3H), 0.82 (s, 3H), 0.81 (d, *J* = 6.7 Hz, 3H). ^13^C NMR (100 MHz, DMSO-*d*_6_) *δ* 141.7, 120.9, 70.5, 70.3, 66.9, 61.5, 54.5, 50.1, 42.7, 42.2, 37.7, 37.3, 36.6, 36.0, 35.9, 33.8, 31.9, 31.8, 31.6, 29.8, 23.9, 20.8, 19.6, 18.5, 17.3, 13.3.

#### 3.1.2. Synthesis of (3*S*,8*S*,9*S*,10*R*,13*S*,14*S*,16*S*,17*R*)-16-Hydroxy-10,13-Dimethyl-17-((2*R*,6*R*)-6-Methyl-7-(Pivaloyloxy)Heptan-2-yl)-2,3,4,7,8,9,10,11,12,13,14,15,16,17-Tetradecahydro-1H-Cyclopenta[a]Phenanthren-3-yl Pivalate (**9**) 

Pivaloyl chloride (0.5 mL, 4.0 mmol) was slowly added to a solution of compound **8** (513 mg, 1.2 mmol) in 10 mL of THF and 5 mL of pyridine at 0 °C with an ice bath, then the ice bath was removed and the mixture was stirred at room temperature overnight. After the reaction was completed (detected by TLC), the solution was worked up by the addition of water (10 mL) and then extracted with EtOAc (20 mL × 3). The combined EtOAc extracts were washed with brine, dried over Na_2_SO_4_, filtered and condensed by rotary evaporation to yield a yellow oil. The residue was purified by silica gel chromatography (gradient: 10% to 20% EtOAc in petroleum ether) and provided product **9** (355 mg, 49%, R_f_ = 0.3 in petroleum ether: EtOAc = 5:1) as a white solid. ^1^H NMR (CDCl_3_, 400 MHz) *δ* 5.30 (d, *J* = 4.5 Hz, 1H), 4.55–4.45 (m, 1H), 4.28 (m, 1H), 3.91 (dd, *J* = 10.7, 5.6 Hz, 1H), 3.74 (dd, *J* = 10.7, 6.9 Hz, 1H), 2.25–2.13 (m, 3H), 1.96–1.87 (m, 2H), 1.82–1.71 (m, 4H), 1.13 (s, 9H), 1.11 (s, 9H), 0.97 (s, 3H), 0.91 (d, *J* = 6.7 Hz, 3H), 0.86 (d, *J* = 6.8 Hz, 3H), 0.82 (s, 3H). ^13^C NMR (CDCl_3_, 100 MHz) *δ* 178.7, 178.1, 139.9, 122.2, 73.5, 72.4, 69.2, 61.4, 54.4, 50.9, 42.2, 39.8, 38.9, 38.6, 38.0, 36.9, 36.7, 36.6, 36.1, 33.7, 32.6, 31.8, 31.5, 29.8, 27.6, 27.2, 27.2, 23.6, 20.7, 19.4, 18.2, 17.0, 13.0.

#### 3.1.3. Synthesis of (2*R*,6*R*)-6-((3*S*,8*S*,9*S*,10*R*,13*S*,14*S*,16*S*,17*R*)-10,13-Dimethyl-16-((Methylsulfonyl)oxy)-3-(Pivaloyloxy)-2,3,4,7,8,9,10,11,12,13,14,15,16,17-Tetradecahydro-1H-Yclopenta[a]Phenanthren-17-yl)-2-Methylheptyl Pivalate (**10**) 

A solution of methanesulfonic anhydride (1.4 g, 8.2 mmol) in 15 mL dry pyridine was added to a solution of compound **9** (1.0 g, 1.6 mmol) in 6 mL dry pyridine at 0 °C with an ice bath. The mixture was stirred at 0 °C to room temperature for overnight. After the reaction was completed (detected by TLC), the solution was worked up by the addition of water (10 mL) and then extracted with EtOAc (20 mL × 3). The combined EtOAc extracts were washed with 1 N HCl, NaHCO_3_ (aq. sat.), brine, dried over Na_2_SO_4_, filtered and condensed by rotary evaporation to yield a yellow oil. The residue was purified by silica gel chromatography (gradient: 10% to 20% EtOAc in petroleum ether) and provided product **10** (1.1 g, 99%, R_f_ = 0.2 in petroleum ether: EtOAc = 5:1) as a white solid. ^1^H NMR (CDCl_3_, 400 MHz) *δ* 5.28 (d, *J* = 5.1 Hz, 1H), 5.17–5.09 (m, 1H), 4.55–4.44 (m, 1H), 3.89–3.83 (m, 1H), 3.82–3.74 (m, 1H), 2.90 (s, 3H), 2.32 (dt, *J* = 14.3, 7.5 Hz, 1H), 2.22 (d, *J* = 8.2 Hz, 2H), 1.98 (d, *J* = 12.1 Hz, 1H), 1.91 (d, *J* = 12.6 Hz, 1H), 1.83–1.65 (m, 5H), 1.56–1.16 (m, 14H), 1.13 (s, 9H), 1.11 (s, 9H), 1.08–1.04 (m, 2H), 0.97 (s, 3H), 0.91 (d, *J* = 6.7 Hz, 3H), 0.85 (d, *J* = 6.8 Hz, 3H), 0.81 (s, 3H). ^13^C NMR (100 MHz, CDCl_3_) *δ* 178.7, 178.0, 139.9, 121.9, 83.2, 73.4, 69.2, 60.7, 54.7, 49.8, 42.4, 39.3, 38.9, 38.9, 38.6, 37.9, 36.9, 36.6, 35.3, 34.8, 33.6, 32.6, 31.6, 31.3, 30.1, 27.6, 27.2, 27.2, 23.8, 20.6, 19.3, 18.0, 16.9, 12.6.

#### 3.1.4. Synthesis of (3*S*,8*S*,9*S*,10*R*,13*R*,14*S*,17*R*)-17-((2*R*,6*R*)-7-Hydroxy-6-Methylheptan-2-yl)-10,13-Dimethyl-2,3,4,7,8,9,10,11,12,13,14,15,16,17-Tetradecahydro-1H-Cyclopenta[a]Phenanthren-3-ol (**11**) 

Compound **10** (1.1 g, 1.6 mmol) was dissolved in 15 mL of dry ether and was added dropwise to a mixture of LiAlH_4_ (623 mg, 16.4 mmol) in 15 mL of dry ether. The mixture was heated to reflux and stirred at reflux for 10 h. After the reaction was completed (detected by TLC), the reaction was quenched with water. The solution was worked up by the addition of 1 N NaOH (4 mL) and then extracted with EtOAc (20 mL × 3). The combined EtOAc extracts were washed with brine, dried over Na_2_SO_4_, filtered and condensed by rotary evaporation to yield a yellow oil. The residue was purified by silica gel chromatography (gradient: 1 to 5% MeOH in CH_2_Cl_2_) and provided product **11** (600 mg, 91%, R_f_ = 0.2 in CH_2_Cl_2_: MeOH = 20:1) as a white solid. ^1^H NMR (400 MHz, DMSO-*d*_6_) *δ* 5.26 (d, *J* = 5.0 Hz, 1H), 4.60 (d, *J* = 4.6 Hz, 1H), 4.35 (t, *J* = 5.3 Hz, 1H), 3.29–3.19 (m, 2H), 3.19–3.11 (m, 1H), 2.18–2.02 (m, 2H), 2.01–1.85 (m, 2H), 1.81–1.71 (m, 2H), 1.67 (d, *J* = 12.2 Hz, 1H), 1.58–0.95 (m, 20H), 0.94 (s, 3H), 0.89 (d, *J* = 6.5 Hz, 3H), 0.80 (d, *J* = 6.6 Hz, 3H), 0.65 (s, 3H). ^13^C NMR (100 MHz, DMSO-*d*_6_) *δ* 141.7, 120.9, 70.5, 66.8, 56.7, 56.1, 50.1, 42.7, 42.3, 37.4, 36.5, 36.2, 35.8, 35.6, 33.7, 31.9, 31.9, 31.8, 29.5, 28.3, 24.4, 23.3, 21.1, 19.6, 19.0, 17.2, 12.2.

#### 3.1.5. Synthesis of (2*R*,6*R*)-6-((3*S*,8*S*,9*S*,10*R*,13*R*,14*S*,17*R*)-3-Hydroxy-10,13-Dimethyl-2,3,4,7,8,9,10,11,12,13,14,15,16,17-Tetradecahydro-1H-Cyclopenta[a]Phenanthren-17-yl)-2-Methylheptyl Acetate (**12**) 

Acetyl chloride (0.1 mL, 2.0 mmol) and Et_3_N (0.4 mL, 3.0 mmol) was added dropwise to a solution of compound **11** (676 mg, 1.7 mmol) and DMAP (24.2 mg, 0.2 mmol) in 20 mL of dry CH_2_Cl_2_ at 0 °C with an ice bath, then the ice bath was removed and the mixture was stirred at room temperature overnight. After the reaction was completed (detected by TLC), the solution was worked up by the addition of water (10 mL) and then extracted with EtOAc (20 mL × 3). The combined EtOAc extracts were washed with 1 N HCl, NaHCO_3_ (aq. sat.), brine, dried over Na_2_SO_4_, filtered and condensed by rotary evaporation to yield a yellow oil. The residue was purified by silica gel chromatography (gradient: 1 to 5% MeOH in CH_2_Cl_2_) and provided product **12** as a colorless oil (314.0 mg, 42% yield, R_f_ = 0.3 in CH_2_Cl_2_: MeOH = 30:1). ^1^H NMR (400 MHz, CDCl_3_) *δ* 5.35 (d, *J* = 5.3 Hz, 1H), 3.97–3.91 (m, 1H), 3.84 (dd, *J* = 10.7, 6.9 Hz, 1H), 3.57–3.48 (m, 1H), 2.06 (s, 3H), 1.01 (s, 3H), 0.92 (s, 3H), 0.91 (s, 3H), 0.68 (s, 3H). ^13^C NMR (101 MHz, CDCl_3_) *δ* 171.4, 140.8, 121.7, 71.8, 69.7, 56.8, 56.1, 50.1, 42.3, 42.3, 39.8, 37.3, 36.5, 36.1, 35.7, 33.8, 32.5, 31.9, 31.7, 29.7, 28.3, 24.3, 23.2, 21.1, 21.0, 19.4, 18.7, 16.8, 11.9.

#### 3.1.6. Synthesis of (2*R*,6*R*)-2-Methyl-6-((3*S*,5*R*,6*R*,8*S*,9*S*,10*R*,13*R*,14*S*,17*R*)-3,5,6-Trihydroxy-10,13-Dimethylhexadecahydro-1H-Cyclopenta[a]Phenanthren-17-yl) Heptyl Acetate (**13**) 

MMPP (485.0 mg, 1.0 mmol) was added to a solution of compound **12** (291.0 mg, 0.6 mmol) in 6 mL of acetone at reflux and the reaction was stirred at reflux for 30 min. Then, the mixture solution was cooled to room temperature, the acetone was evaporated, the residue was dissolved in 20 mL of CH_2_Cl_2_ and the mixture solution was washed with water and brine and dried over anhydrous Na_2_SO_4_, filtered and condensed by rotary evaporation. The residue was dissolved in 6 mL of acetone, Bi(OTf)_3_ (46.0 mg, 0.1 mmol) was added and the mixture was stirred at room temperature for 1 h. After the reaction was completed (detected by TLC), the solution was worked up by the addition of water (10 mL) and then extracted with EtOAc (20 mL × 3). The combined EtOAc extracts were washed with brine, dried over Na_2_SO_4_, filtered and condensed by rotary evaporation to yield a yellow oil. The residue was purified by silica gel chromatography (gradient: 1 to 5% MeOH in CH_2_Cl_2_) and provided product **13** (127.0 mg, 41%, R_f_ = 0.2 in CH_2_Cl_2_: MeOH = 20:1) as a white solid. M.p. 201–202 °C. ^1^H NMR (400 MHz, DMSO-*d*_6_) *δ* 4.39 (d, *J* = 4.1 Hz, 1H), 4.17 (d, *J* = 5.5 Hz, 1H), 3.86 (dd, *J* = 10.7, 6.0 Hz, 1H), 3.78 (dd, *J* = 10.7, 6.6 Hz, 2H), 3.63 (s, 1H), 3.30 (d, *J* = 3.8 Hz, 1H), 2.00 (s, 3H), 1.95–1.82 (m, 2H), 1.80–1.66 m, 2H), 1.62–1.42 (m, 5H), 1.41–1.03 (m, 17H), 1.02 (s, 3H), 0.98 (m, 2H),0.88 (d, *J* = 6.3 Hz, 3H), 0.86 (d, *J* = 6.7 Hz, 3H), 0.63 (s, 3H). ^13^C-NMR (101 MHz, DMSO-*d*_6_) *δ* 170.9, 74.8, 74.6, 69.1, 66.2, 56.3, 56.2, 45.0, 42.8, 41.4, 38.3, 36.0, 35.6, 35.0, 33.5, 32.5, 32.4, 31.6, 30.5, 28.3, 24.4, 23.1, 21.2, 21.2, 19.0, 17.0, 16.8, 12.4. HRMS (ESI) [M + NH_4_]^+^ *m/z* calcd for C_29_H_54_O_5_N 496.3997, found: 476.3987.

#### 3.1.7. Synthesis of (2*R*,6*R*)-6-((3*S*,5*R*,8*S*,9*S*,10*R*,13*R*,14*S*,17*R*)-3,5-Dihydroxy-10,13-Dimethyl-6-Oxohexadecahydro-1H-Cyclopenta[a]Phenanthren-17-yl)-2-Methylheptyl Acetate ((25*R*)-5) 

*N*-bromosuccinimide (79.0 mg, 0.4 mmol) was added in three portions to a mixture solution of compound **13** (105.0 mg, 0.2 mmol) in acetone (4 mL), acetic acid (15.0 μL, 0.3 mmol) and H_2_O (180.0 μL) at 0 °C with an ice bath, then the ice bath was removed and the mixture was stirred at room temperature for 1 h. After the reaction was completed (detected by TLC), the reaction solution was quenched by the addition of Na_2_SO_3_ (aq. sat.). The solution was extracted with EtOAc (20 mL × 3). The combined EtOAc extracts were washed with brine, dried over Na_2_SO_4_, filtered and condensed by rotary evaporation to yield a yellow oil. The residue was purified by silica gel chromatography (gradient: 1 to 5% MeOH in CH_2_Cl_2_) provided product (25*R*)-**5** (49.0 mg, 47%, R_f_ = 0.2 in CH_2_Cl_2_: MeOH = 20:1) as a white solid. [α]^20^_D_ − 14.6 (*c* 0.83, MeOH). mp 202–203 °C. ^1^H NMR (400 MHz, CDCl_3_) *δ* 4.01–3.98 (m, 1H), 3.93 (dd, *J* = 10.6, 6.0 Hz, 1H), 3.83 (dd, *J* = 10.7, 6.9 Hz, 1H), 2.73 (t, *J* = 12.6 Hz, 1H), 2.10 (dd, *J* = 13.0, 4.6 Hz, 1H), 2.05 (s, 3H), 2.03–1.98 (m, 1H), 1.89–1.82 (m, 4H), 1.79–1.66 (m, 4H), 1.56–1.42 (m, 4H), 1.38–1.20 (m, 9H), 1.15–1.10 (m, 2H), 1.07–0.95 (m, 2H), 0.91 (d, *J* = 6.7 Hz, 3H), 0.90 (d, *J* = 6.5 Hz, 3H), 0.79 (s, 3H), 0.63 (s, 3H). ^13^C NMR (100 MHz, CDCl_3_) *δ* 213.5, 171.4, 80.5, 69.6, 67.2, 56.3, 56.1, 44.3, 43.1, 42.5, 41.8, 39.6, 37.4, 36.1, 36.0, 35.6, 33.7, 32.5, 30.2, 29.8, 28.1, 23.9, 23.3, 21.4, 21.0, 18.6, 16.8, 14.0, 12.0. ^1^H NMR (400 MHz, DMSO-*d*_6_) *δ* 5.29 (s, 1H), 4.35 (d, *J* = 5.6 Hz, 1H), 3.86 (dd, *J* = 10.7, 6.0 Hz, 1H), 3.78 (dd, *J* = 10.7, 6.7 Hz, 1H), 3.70 (m, 1H), 2.68 (t, *J* = 12.5 Hz, 1H), 2.00 (s, 3H), 1.95 (dd, *J* = 8.8, 2.7 Hz, 1H), 1.85 (d, *J* = 4.2 Hz, 1H), 1.82 (d, *J* = 4.3 Hz, 1H), 1.73–0.95 (m, 24H), 0.89 (d, *J* = 6.4 Hz, 3H), 0.86 (d, *J* = 6.6 Hz, 3H), 0.66 (s, 3H), 0.61 (s, 3H). ^13^C NMR (100 MHz, DMSO-*d*_6_) *δ* 212.7, 170.8, 79.6, 69.1, 65.7, 56.4, 56.0, 44.2, 43.1, 42.4, 41.9, 37.3, 36.2, 36.0, 35.6, 33.6, 32.4, 30.9, 30.0, 28.2, 24.0, 23.2, 21.5, 21.1, 18.9, 17.0, 14.0, 12.3. HRMS (ESI) [M + H]^+^ *m/z* calcd for C_29_H_49_O_5_ 477.3580, found: 477.3578; [M + Na]^+^ *m*/*z* calcd for C_29_H_48_O_5_Na 499.3399, found: 499.3401.

### 3.2. Biological Studies

#### 3.2.1. Anti-RSV Activity Assay

The anti-RSV activities of compounds (25*R*)-**5**, **8**, **11**, **13** and **5** were determined by using a RT-qPCR method in HEp-2 cells, according to established procedures [28]. Remdesivir was selected and evaluated as a positive control.

#### 3.2.2. Anti-Proliferation Assays

The anti-proliferation activities of compounds (25*R*)-**5**, **8**, **11** and **13** against nineteen human cancer cell lines and a normal liver cell line were explored by using a CCK-8 assay [29,30,31,32,33]. One concentration was set for each sample during preliminary screening and three multiple holes were set for each concentration. Eight concentration gradients were set for each sample for IC_50_ determination and three multiple holes were set for each concentration. The 96-well plates were cultured at 5% CO_2_ and 37 °C for 48 h. The old culture medium with a drug solution of adherent cells was sucked out, then 100 μL of CCK-8 solution (diluted ten times with the basic medium) was added and the suspension cells was directly added 10 μL of CCK-8 stock solution, cultured at 37 °C with 5% CO_2_ for 1–4 h (dark operation, real-time observation). The absorbance was measured at 450 nm with an enzyme labeling instrument and the original data and results were recorded. The IC_50_ was calculated by software GraphPad prism 8 (version 8.0.2, from GraphPad Software Inc., San Diego, CA, USA) and the experimental results were expressed in ± SD. Doxorubicin was used as a positive control. All the cancer cell lines were obtained from Qingdao AC biotechnology Co., LTD, Qingdao, China.

#### 3.2.3. Apoptosis Assays

The percent of apoptosis in cells was assayed according to the instructions (Vazyme, A211). 5637 (HTB-9) cells were seeded in 6-well plates at 2 × 10^5^ cells/well overnight and treated with compound (25*R*)-**5** at various concentrations for 48 h. Trypsinization of the cells occurred without EDTA and they were collected in tubes and suspended in 100 μL ice-cold 1 × binding buffer after being washed twice with PBS. Cells were stained with PI and FITC Annexin V for 10 min at room temperature following the instructions of the Annexin V FITC/PI kit (Vazyme Biotech, Nanjing, China). Next, 200 μL ice-cold 1 × binding buffer was added. The result was measured by flow cytometry (MoFlo XDP, Beckman, Pasadena, CA, USA). For each condition, a minimum of 10,000 cells were analyzed. Triplicate experiments were performed independently.

## 4. Conclusions

In conclusion, we have first efficiently semi-synthesized the single isomer of steroid compound **5**, also named as (25*R*)-**5**, in seven steps from a commercially available nature product diosgenin (**7**), with a total yield of 2.8%. The structure and absolute configuration of compound (25*R*)-**5** was confirmed by HRMS, ^1^H and ^13^C NMR and HPLC analysis. Furthermore, compound (25*R*)-**5**, together with the important intermediates **8**, **11** and **13,** was evaluated for the anti-viral activities against RSV, but none of them showed potent inhibition of RSV replication at the concentration of 10 µM. These compounds did not exhibit sufficient activity for further anti-RSV studies, but they could be seen as good leads for further structure optimization due to their derivates (**1**–**4**) having potent anti-RSV activities. Intriguingly, these four compounds displayed promising anti-proliferative activities against several cancer cell lines without affecting the normal human liver L-02 cell line. The initial SAR studies indicated that the hydroxyl group at the C-16 position and the double-bond of compound **8** had important roles for the anti-proliferation activity against these cancer cell lines. Results of cell flow cytometry indicated that compound (25*R*)-**5** could exert the cell-mediated cytotoxicity with the mechanism of inducing cancer cell apoptosis. These studies suggested that compound **5** has great therapeutic potential as an anti-tumor agent. Further structure optimization and SAR study will be reported in due course.

## Figures and Tables

**Table 1:**
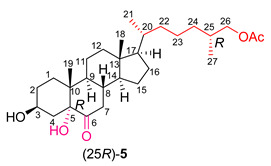
^1^H NMR and ^13^C NMR data of compound **5** and (25*R*)-**5**.

Position	5 (CDCl_3_, *δ* in ppm) ^a^		(25*R*)-5 (CDCl_3_, *δ* in ppm) ^b^	
	^1^H NMR	^13^C NMR	^1^H NMR	^13^C NMR
1	1.70, m 1.54, brd	29.8	1.79–1.66, m 1.56–1.42, m	29.8
2	1.87, m 1.46, m	30.3	1.89–1.82, m 1.56–1.42, m	30.2
3	3.98, m	67.3	4.01–3.98, m	67.2
4	1.86–1.81, m 1.79, m	36.4	1.89–1.82, m 1.79–1.66, m	36.1
5		80.9		80.5
6		212.3		213.5
7	2.72, dd (13.2, 12.2) 2.12, dd (13.2, 4.4)	41.8	2.73, t (12.6) 2.10, dd (13.0, 4.6)	41.8
8	1.70, m	37.3	1.79–1.66, m	37.4
9	1.86–1.81, m	44.5	1.89–1.82, m	44.3
10		42.4		42.5
11	1.46, m 1.29, m	21.4	1.56–1.42, m 1.38–1.20, m	21.4
12	2.02, ddd (12.2, 3.4, 2.4) 1.23, ddd (12.8, 12.2, 3.4)	39.6	2.03–1.98, m 1.38–1.20, m	39.6
13		43.1		43.1
14	1.31, m	56.3	1.38–1.20, m	56.3
15	1.51, m 1.04, m	23.9	1.56–1.42, m 1.07–0.95, m	23.9
16	1.86–1.81, m 1.25, m	28.1	1.89–1.82, m 1.38–1.20, m	28.1
17	1.12, m	56.05 56.02	1.15–1.10, m	56.1
18	0.65, s	12.0	0.64, s	12.0
19	0.81, s	14.1	0.79, s	14.0
20	1.35, m	35.68 35.62	1.38–1.20, m	35.6
21	0.91, d (6.8)	18.6	0.90, d (6.5)	18.6
22	1.30, m 1.21, m	33.85 33.70	1.38–1.20, m	33.7
23	1.35, m 1.19, m	23.30 23.26	1.38–1.20, m	23.3
24	1.35, m 1.02, m	36.05 35.95	1.38–1.20, m 1.07–0.95, m	36.0
25	1.77, m	32.54 32.48	1.79–1.66, m	32.5
26	0.93, d (6.8), maj 0.92, d (6.8), maj	17.01 16.78	0.91, d (6.7)	16.8
27	3.96, dd (10.7, 7.3), min 3.95, dd (10.7, 7.3), maj 3.84, dd (10.7, 2.4), maj 3.83, dd (10.7, 1.9), min	69.59 69.45	3.93, dd, (10.6, 6.0) 3.83, dd, (10.7, 6.9)	69.6
OA_C_	2.06, s	171.3 (C) 21.0 (CH_3_)	2.05, s	171.4 (C) 21.0 (CH_3_)

^a^ Data are taken from reference [19]. ^b^ NMR spectra were recorded on a BRUKER AVANCE NEO (^1^H, 400 MHz; ^13^C, 100 MHz) spectrometer. ^1^H and ^13^C NMR spectra were recorded with TMS as an internal reference.

**Table 2:** Anti-RSV activities of compounds (25*R*)-**5**, **8**, **11**, **13** and **5** ^a^.

Compounds	8	11	13	(25*R*)-5	5	Remdesivir
Inhibition rate (%)	24.9 ± 2.6	22.2 ± 3.9	13.6 ± 2.1	29.4 ± 7.1	18.3 ± 3.8	91.8 ± 0.5

^a^ The values are the mean ± SEM of at least three independent experiments. The inhibition rates (%) were calculated by comparing the untreated cells.

**Table 3:** Anti-cancer activities of compounds (25*R*)-**5**, **8**, **11** and **13** ^a^.

	Compds.	Inhibition Rate (%)				
Cell Lines		8 (at 20 µM)	11 (at 20 µM)	13 (at 20 µM)	(25*R*)-5 (at 20 µM)	Doxorubicin (at 10 µM)
K-562		7.2 ± 2.2	13.1 ± 1.8	7.8 ± 2.3	17.1 ± 2.8	67.5 ± 1.0
A549		53.0 ± 2.7	31.2 ± 3.9	3.9 ± 2.7	35.3 ± 1.3	75.9 ± 2.5
MKN-45		54.4 ± 1.9	53.8 ± 1.6	52.7 ± 2.4	61.1 ± 1.4	89.8 ± 0.5
HCT 116		61.7 ± 2.1	19.8 ± 1.4	19.3 ± 1.2	59.3 ± 3.8	88.9 ± 1.5
HeLa		49.7 ± 3.6	14.9 ± 2.2	−2.3 ± 1.7	22.7 ± 1.2	97.7 ± 0.4
786-O		69.0 ± 1.8	65.9 ± 2.4	20.5 ± 1.3	65.3 ± 0.5	86.3 ± 1.9
TE-1		34.0 ± 3.8	27.7 ± 0.6	13.5 ± 2.6	29.5 ± 2.2	83.3 ± 1.1
5637 (HTB-9)		75.1 ± 0.8	80.5 ± 2.5	52.2 ± 4.5	64.7 ± 3.4	99.3 ± 0.3
GBC-SD		26.8 ± 1.3	10.4 ± 3.3	6.0 ± 2.4	28.1 ± 1.5	76.7 ± 1.5
L-02		8.1 ± 1.3	2.8 ± 2.1	6.3 ± 3.2	−4.9 ± 3.4	99.2 ± 0.1
MCF7		31.2 ± 1.4	14.1 ± 0.5	19.0 ± 1.1	19.3 ± 2.1	55.9 ±1.7
HepG2		76.8 ± 1.3	64.2 ± 2.5	58.0 ± 1.2	66.8 ± 2.1	95.9 ±1.9
SF126		30.7 ± 2.4	11.7 ± 2.9	4.7 ± 1.3	28.1 ± 0.5	97.1 ± 1.0
DU145		26.0 ± 1.7	18.4 ± 3.7	−0.4 ± 3.9	12.2 ± 2.0	76.4 ± 1.6
CAL-62		28.0 ± 4.0	12.5 ± 3.9	7.5 ± 2.7	18.2 ± 1.9	92.6 ± 1.3
PATU8988T		62.7 ± 2.0	63.3 ± 4.2	53.6 ± 1.7	65.9 ± 4.2	98.4 ± 0.6
HOS		2.7 ± 4.0	9.3 ± 4.3	52.6 ± 1.5	22.2 ± 1.1	98.4 ± 0.9
A-375		72.3 ± 0.6	60.8 ± 1.4	54.8 ± 1.7	76.3 ± 1.7	95.3 ± 1.0
A-673		51.9 ± 2.8	26.5 ± 2.1	66.3 ± 3.2	49.4 ± 1.4	98.7 ± 0.6
293T		42.4 ± 2.1	−18.2 ± 1.3	37.3 ± 2.7	26.2 ± 1.5	88.2 ± 1.2

^a^ The values are the mean ± SEM of at least three independent experiments. The inhibition rates (%) were calculated by the calculation formula as follows: inhibition rate (%) = (OD_Control_ − OD_Compound_)/(OD_Control_ − OD_Blank_) × 100%. K-562 (human myeloid leukemia cell); A549 (human lung adenocarcinoma cell); MKN-45 (human gastric cancer cell); HCT 116 (human colorectal cancer cell); HeLa (human cervical cancer cell); 786-O (human renal cancer cell); TE-1 (human esophageal cancer cell); 5637 (HTB-9) (human bladder cancer cell); GBC-SD (human gallbladder carcinoma cell); L-02 (human hepatocyte cell); MCF7 (human breast cancer cell); HepG2 (human liver carcinoma cell); SF126 (human glioblastoma cell); DU145 (human prostate carcinoma cell); CAL-62 (human thyroid anaplastic carcinoma cell); PATU8988T (human pancreatic cancer cell); HOS (human osteosarcoma cell); A-375 (human melanoma cell); A-673 (Ewing sarcoma); 293T (human embryonic kidney cell).

## Data Availability

The data presented in this study are available in the manuscript and in the Supplementary Materials.

## Supplementary Materials

The following supporting information can be downloaded at: https://www.mdpi.com/article/10.3390/md21030191/s1, Figure S1: High-resolution electron spray ionization mass (HRMS-ESI) of compound (25*R*)-**5**; Figure S2: The copy of ^1^H NMR spectrum (400 MHz, CDCl_3_) of (25*R*)-**5**. Figure S3: The copy of ^13^C NMR spectrum (100 MHz, CDCl_3_) of (25*R*)-**5**. Figure S4: The copy of ^1^H NMR spectrum (400 MHz, DMSO-*d*_6_) of (25*R*)-**5**. Figure S5: The copy of ^13^C NMR spectrum (100 MHz, DMSO-*d*_6_) of (25*R*)-**5**. Figure S6: HPLC chromatograms of compounds (25*R*)-**5** and **5**. (A) HPLC chromatogram of compound (25R)-**5**. (B) HPLC chromatogram of compound **5**.

## References

[1] Bhatti H.N., Khera R.A. (2012). Biological transformations of steroidal compounds: A review. Steroids.

[2] Sharma K., Kumar H., Priyanka (2022). Formation of nitrogen-containing six-membered heterocycles on steroidal ring system: A review. Steroids.

[3] Huo H., Li G., Shi B., Li J. (2022). Recent advances on synthesis and biological activities of C-17 aza-heterocycle derived steroids. Bioorg. Med. Chem..

[4] Bansal R., Acharya P.C. (2014). Man-made cytotoxic steroids: Exemplary agents for cancer therapy. Chem. Rev..

[5] Dabbah-Assadi F., Handel R., Shamir A. (2022). What we know about the role of corticosteroids in psychiatric disorders; evidence from animal and clinical studies. J. Psychiatr. Res..

[6] Guevara M.A., Lu J., Moore R.E., Chambers S.A., Eastman A.J., Francis J.D., Noble K.N., Doster R.S., Osteen K.G., Damo S.M. (2020). Vitamin D and streptococci: The interface of nutrition, host Immune response, and antimicrobial activity in response to infection. ACS Infect. Dis..

[7] Thomas C., Pellicciari R., Pruzanski M., Auwerx J., Schoonjans K. (2008). Targeting bile-acid signalling for metabolic diseases. Nat. Rev. Drug Discov..

[8] Plum L.A., DeLuca H.F. (2010). Vitamin D, disease and therapeutic opportunities. Nat. Rev. Drug Discov..

[9] Hurley M.J., Bates R., Macnaughtan J., Schapira A.H.V. (2022). Bile acids and neurological disease. Pharmacol. Ther..

[10] Madasu C., Xu Y.-M., Wijeratne E.M.K., Liu M.X., Molnár I., Gunatilaka A.A.L. (2022). Semi-synthesis and cytotoxicity evaluation of pyrimidine, thiazole, and indole analogues of argentatins A–C from guayule (*Parthenium argentatum*) resin. Med. Chem. Res..

[11] Xu Y.-m., Madasu C., Liu M.X., Wijeratne E.M.K., Dierig D., White B., Molnár I., Gunatilaka A.A.L. (2021). Cycloartane- and Lanostane-Type Triterpenoids from the Resin of Parthenium argentatum AZ-2, a Byproduct of Guayule Rubber Production. ACS Omega.

[12] Tong W.Y., Dong X. (2009). Microbial biotransformation: Recent developments on steroid drugs. Recent Pat. Biotechnol..

[13] Gundamraj S., Hasbun R. (2020). The Use of Adjunctive Steroids in Central Nervous Infections. Front. Cell. Infect. Microbiol..

[14] Chen B., Gu Y.C., Voogd N.J., Wang C.Y., Guo Y.W. (2022). Xidaosterols A and B, two new steroids with unusual alpha-keto-enol functionality from the south China sea sponge neopetrosia chaliniformis. Nat. Prod. Res..

[15] Chen B., Li W.S., Gu Y.C., Zhang H.Y., Luo H., Wang C.Y., Guo Y.W. (2021). New sterols from the south China sea sponges halichondria sp. Fitoterapia.

[16] Cao F., Shao C.L., Chen M., Zhang M.Q., Xu K.X., Meng H., Wang C.Y. (2014). Antiviral C-25 epimers of 26-acetoxy steroids from the south China sea gorgonian echinogorgia rebekka. J. Nat. Prod..

[17] Zhang Y.H., Zhao Y.J., Qi L., Du H.F., Cao F., Wang C.Y. (2022). Talasteroid, a new withanolide from the marine-derived fungus talaromyces stollii. Nat. Prod. Res..

[18] Sun X.P., Cao F., Shao C.L., Chen M., Liu H.J., Zheng C.J., Wang C.Y. (2015). Subergorgiaols A-L, 9,10-secosteroids from the South China Sea gorgonian Subergorgia rubra. Steroids.

[19] Cao F., Shao C.-L., Wang Y., Xu K.-X., Qi X., Wang C.-Y. (2014). Polyhydroxylated Sterols from the South China Sea Gorgonian Verrucella umbraculum. Helv. Chim. Acta.

[20] Chen M., Wu X.D., Zhao Q., Wang C.Y. (2016). Topsensterols A-C, Cytotoxic Polyhydroxylated Sterol Derivatives from a Marine Sponge Topsentia sp. Mar. Drugs.

[21] Battles M.B., McLellan J.S. (2019). Respiratory syncytial virus entry and how to block it. Nat. Rev. Microbiol..

[22] Atanasov A.G., Zotchev S.B., Dirsch V.M., Supuran C.T. (2021). Natural products in drug discovery: Advances and opportunities. Nat. Rev. Drug Discov..

[23] Rho J.-R., Lee H.-S., Seo Y., Cho K.W., Shin J. (2000). New bioactive steroids from the gorgonian *acalycigorgia inernis*. Bull. Korean Chem. Soc..

[24] Gong H., Williams J.R. (2006). Synthesis of the aglycone of the shark repellent pavoninin-4 using remote functionalization. Org. Lett..

[25] Martin R., Schmidt A.W., Theumer G., Krause T., Entchev E.V., Kurzchalia T.V., Knölker H.J. (2009). Synthesis and biological activity of the (25R)-cholesten-26-oic acids--ligands for the hormonal receptor DAF-12 in Caenorhabditis elegans. Org. Biomol. Chem..

[26] Carvalho J.F.S., Silva M.M.C., Moreira J.N., Simões S., Sá e Melo M.L. (2010). Sterols as anticancer agents: Synthesis of ring-B oxygenated steroids, cytotoxic profile, and comprehensive SAR analysis. J. Med. Chem..

[27] Carvalho J.F.S., Silva M.M.C., Sá e Melo M.L. (2010). Efficient trans-diaxial hydroxylation of Δ5-steroids. Tetrahedron.

[28] Joshi S., Chaudhari A.A., Dennis V., Kirby D.J., Perrie Y., Singh S.R. (2018). Anti-RSV peptide-loaded liposomes for the inhibition of respiratory syncytial virus. Bioengineering.

[29] Tominaga H., Ishiyama M., Ohseto F., Sasamoto K., Hamamoto T., Suzuki K., Watanabe M. (1999). A water-soluble tetrazolium salt useful for colorimetric cell viability assay. Anal. Commun..

[30] Liu X., Wei W., Wang C., Yue H., Ma D., Zhu C., Ma G., Du Y. (2011). Apoferritin-camouflaged Pt nanoparticles: Surface effects on cellular uptake and cytotoxicity. J. Mater. Chem..

[31] Lin S.Z., Wei W.T., Chen H., Chen K.J., Tong H.F., Wang Z.H., Ni Z.L., Liu H.B., Guo H.C., Liu D.L. (2012). Antitumor activity of emodin against pancreatic cancer depends on its dual role: Promotion of apoptosis and suppression of angiogenesis. PLoS ONE.

[32] Li X., Zheng S.L., Li X., Li J.L., Qiang O., Liu R., He L. (2012). Synthesis and anti-breast cancer activity of new indolylquinone derivatives. Eur. J. Med. Chem..

[33] Xu M.Y., Lee S.Y., Kang S.S., Kim Y.S. (2014). Antitumor activity of jujuboside B and the underlying mechanism via induction of apoptosis and autophagy. J. Nat. Prod..

