# Supplementary material for: Semi-Synthesis and Biological Evaluation of 25(R)-26-Acetoxy-3β,5α-Dihydroxycholest-6-One
Source: Mar Drugs. 2023 Mar 20;21(3):191. doi: 10.3390/md21030191 (PMC10053440; doi:10.3390/md21030191)
Supplement: Supplementary file 1 [file marinedrugs-21-00191-s001.zip › marinedrugs-2278797-supplementary.pdf]

# SUPPLEMENTARY INFORMATION

## Semi-Synthesis and Biological Evaluation Studies of 25(*R*)-26-Acetoxy-3*β*,5*α*-dihydroxycholest-6-one

**Mireguli Maimaitiming [1,2,†], Ling Lv [1,2,†], Xuetao Zhang [1,2], Shuli Xia [1,2], Xin Li [1,2], Pingyuan Wang [1,2,*], Zhiqing Liu [1,2,*] and Chang-Yun Wang [1,2,*]**

[1] Institute of Evolution & Marine Biodiversity; School of Medicine and Pharmacy; College of Food Science and Engineering, Ocean University of China, Qingdao 266003, China
[2] Laboratory for Marine Drugs and Bioproducts, Qingdao National Laboratory for Marine Science and Technology, Qingdao 266237, China
* Correspondence: wangpingyuan@ouc.edu.cn (P.W.); liuzhiqing@ouc.edu.cn (Z.L.); changyun@ouc.edu.cn (C.-Y.W.)
† These authors contributed equally to this work and should be considered as co-first authors.

### Table of Contents

HRMS-ESI of compound (25*R*)-**5** S1
$^{1}$H and $^{13}$C NMR spectra of compound (25*R*)-**5** S2-S3
$^{1}$H and $^{13}$C NMR spectra of compound **8** S4
$^{1}$H and $^{13}$C NMR spectra of compound **9** S5
$^{1}$H and $^{13}$C NMR spectra of compound **10** S6
$^{1}$H and $^{13}$C NMR spectra of compound **11** S7
$^{1}$H and $^{13}$C NMR spectra of compound **12** S8
$^{1}$H and $^{13}$C NMR spectra of compound **13** S9
HPLC chromatograms of compounds (25*R*)-**5** and **5**. S10
$^{1}$H NMR spectra of diosgenin S11

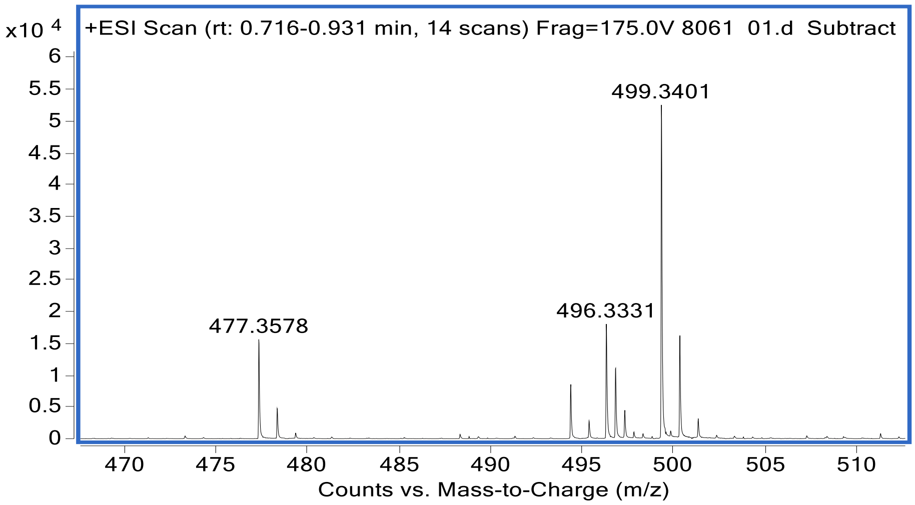


**Figure S1.** High-resolution electron spray ionization mass (HRMS-ESI) of compound (25*R*)-**5.**

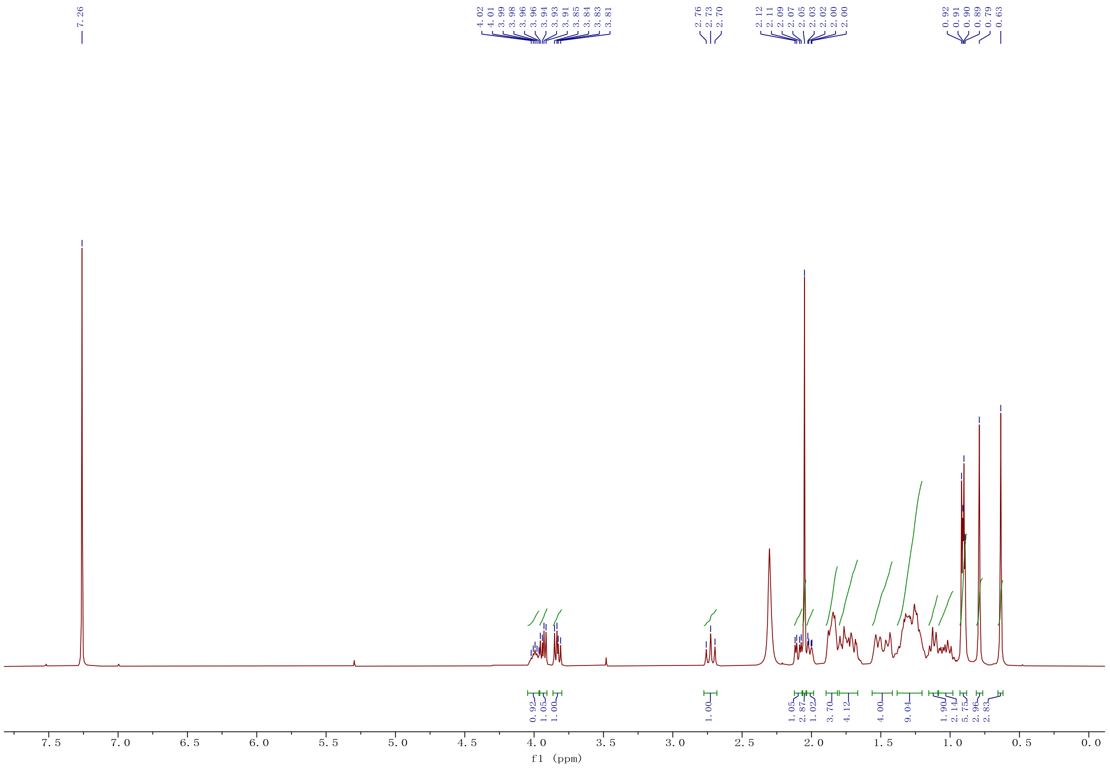


**Figure S2.** The copy of $^{1}$H NMR spectrum (400 MHz, $CDCl_3$) of (25*R*)-**5**.

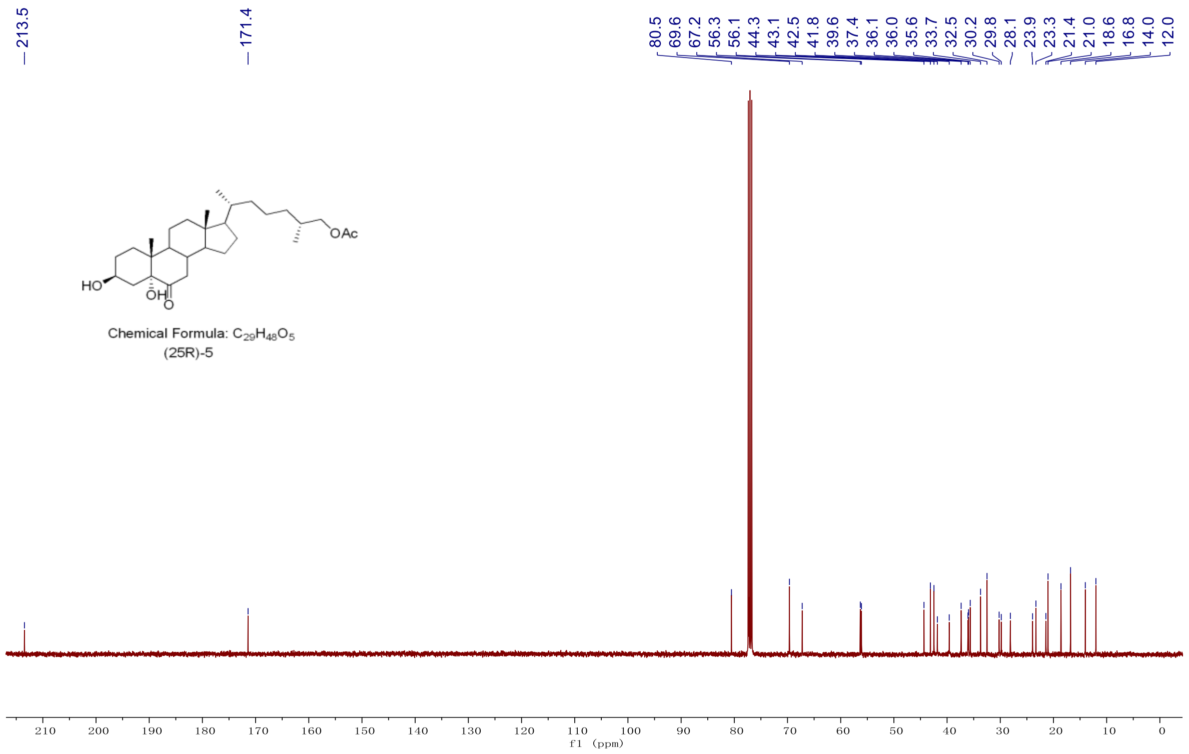


**Figure S3.** The copy of $^{13}$C NMR spectrum (101 MHz, $CDCl_3$) of (25*R*)-**5**.

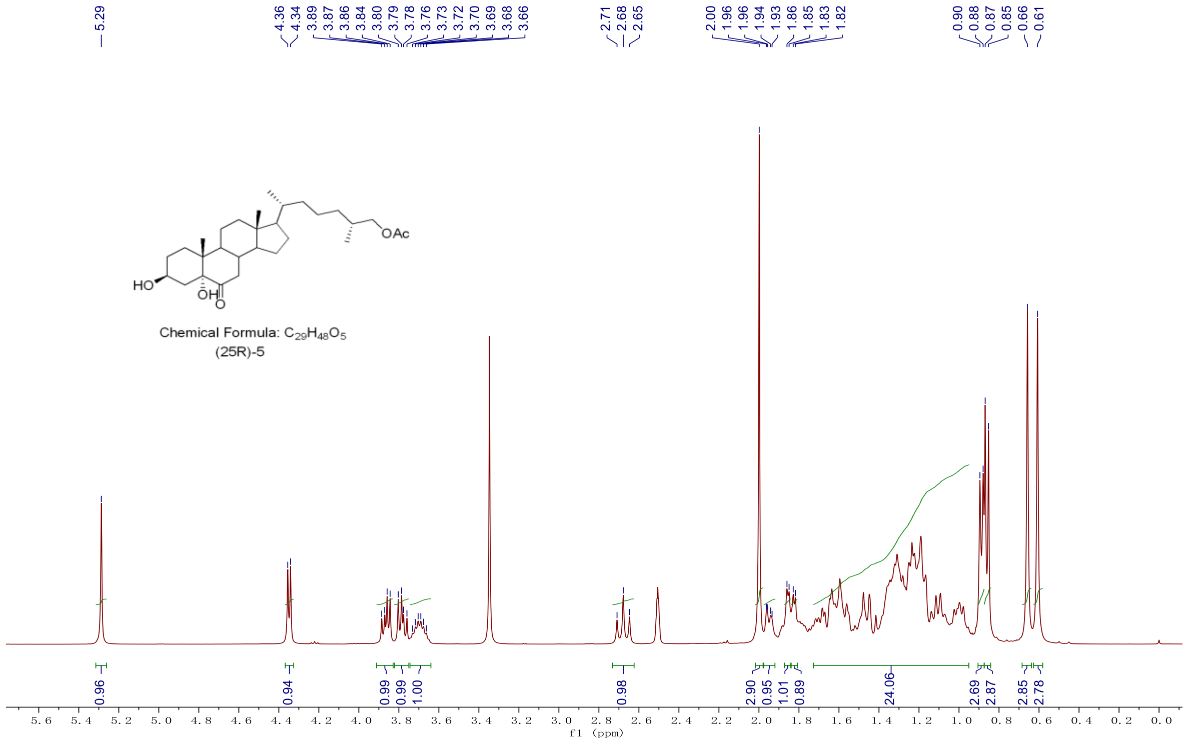


**Figure S4.** The copy of $^{1}$H NMR spectrum (400 MHz, DMSO-*d*6) of (25*R*)-**5**.

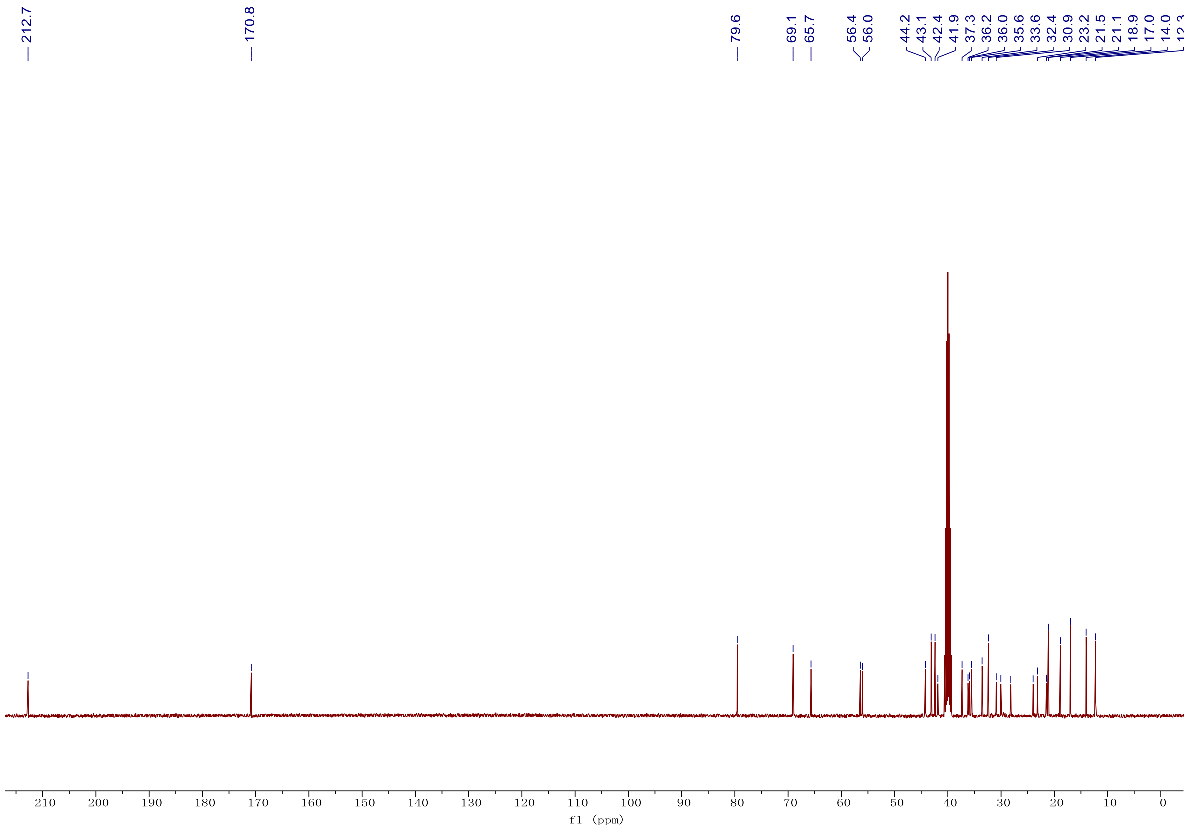

**Figure S5.** The copy of $^{13}$C NMR spectrum (101 MHz, DMSO-*d*6) of (25*R*)-**5**.

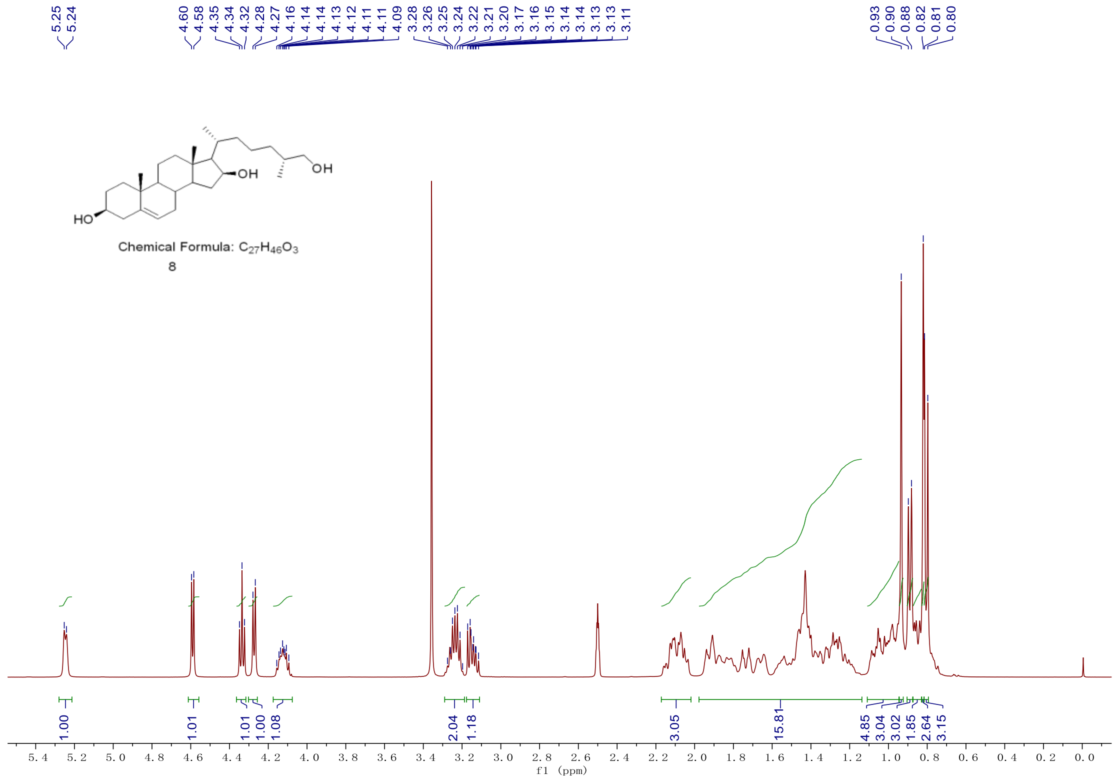


**$^{1}$H NMR spectrum (400 MHz, DMSO-*d*6) of compound 8.**

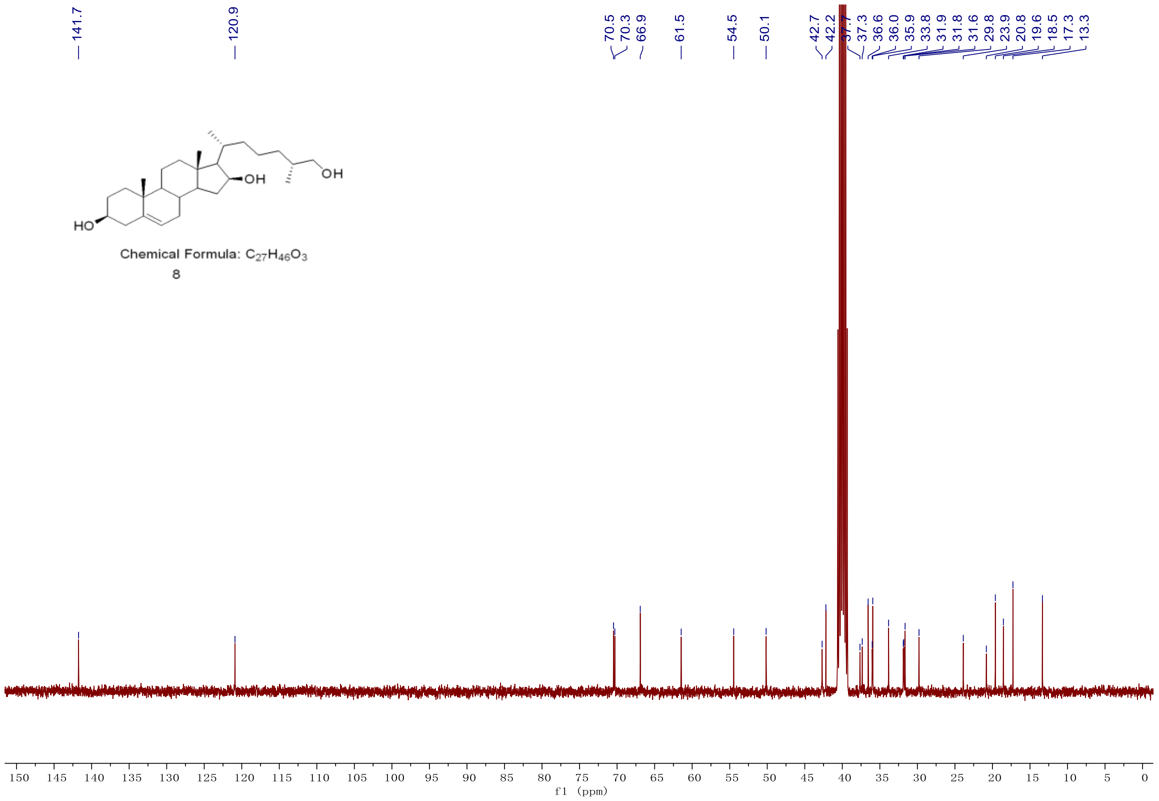


**$^{13}$C NMR spectrum (101 MHz, DMSO-*d*6) of compound 8.**

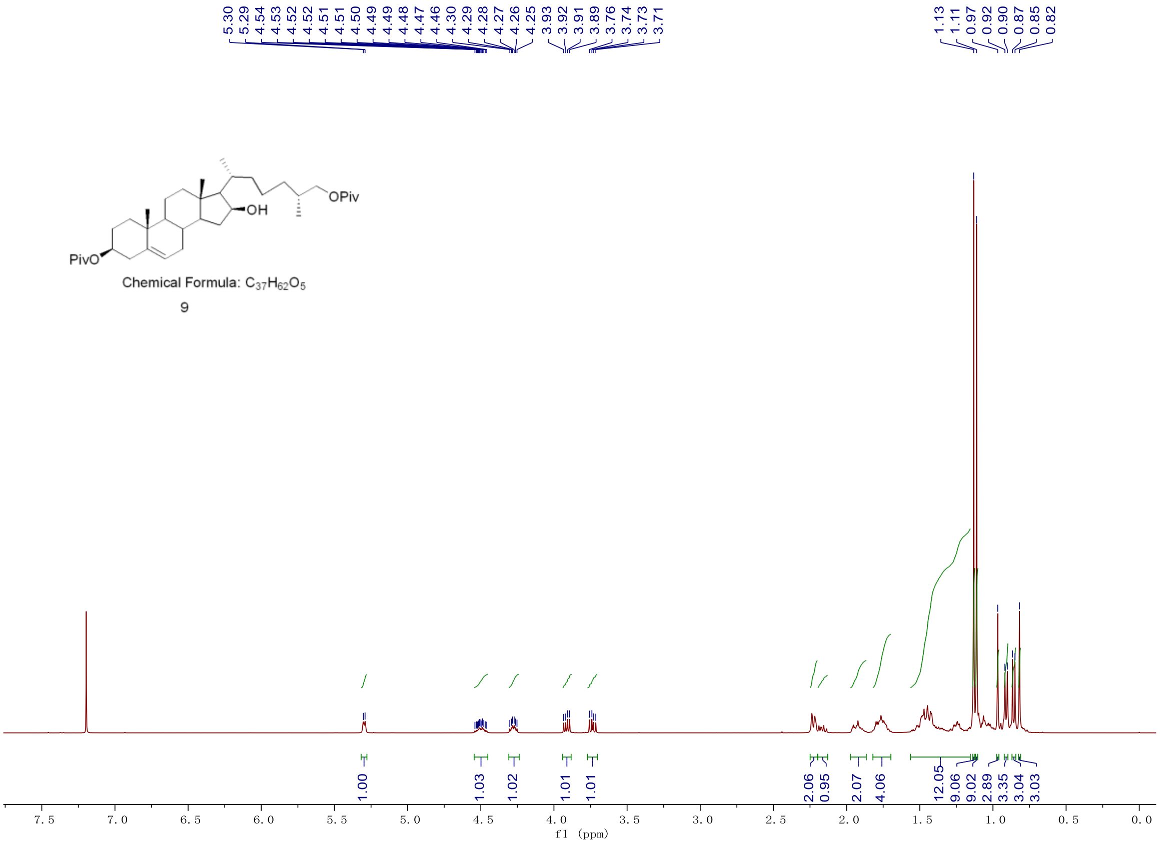


**$^1$H NMR spectrum (400 MHz, $CDCl_3$) of compound 9.**

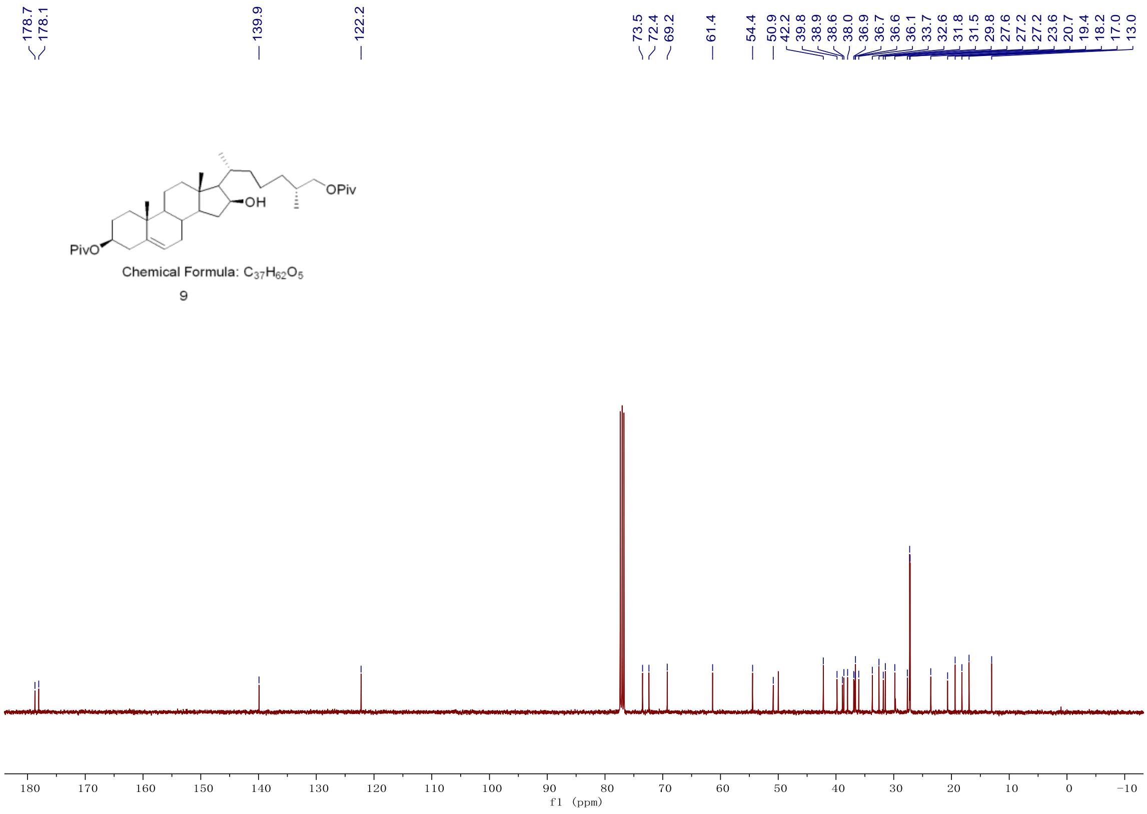


**$^{13}$C NMR spectrum (101 MHz, $CDCl_3$) of compound 9.**

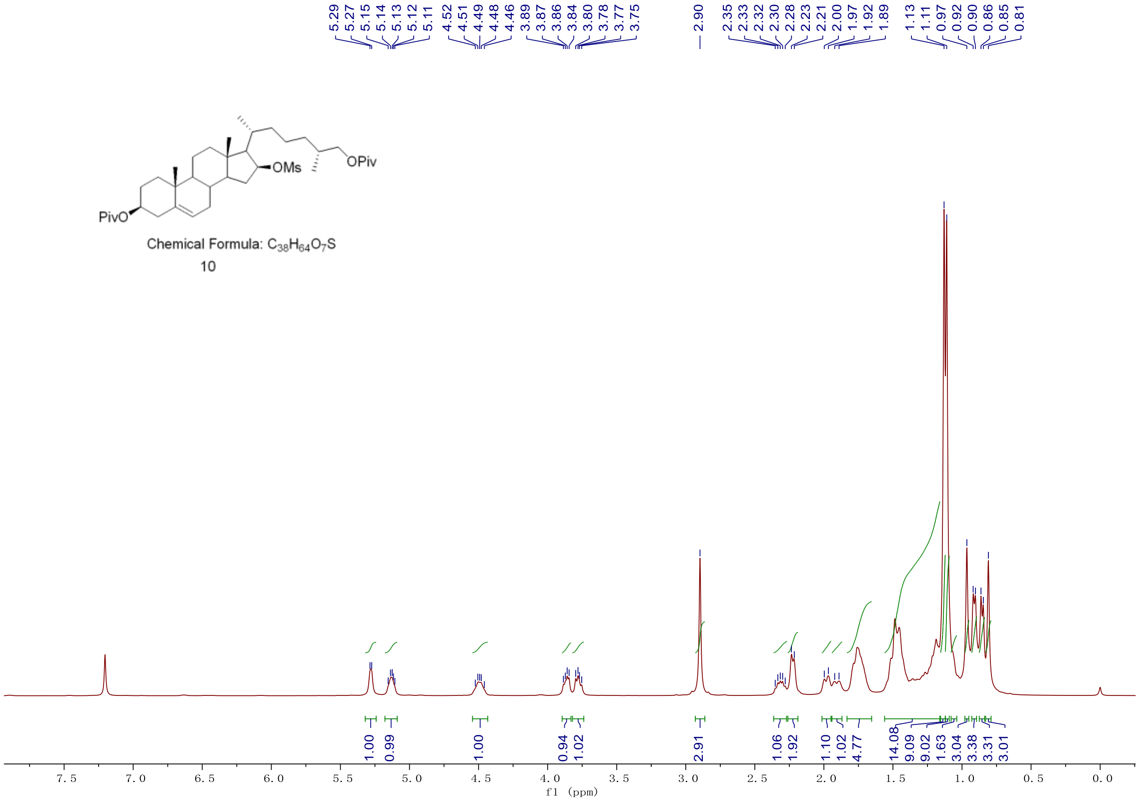


**$^{1}$H NMR spectrum (400 MHz, CDCl$_3$) of compound 10.**

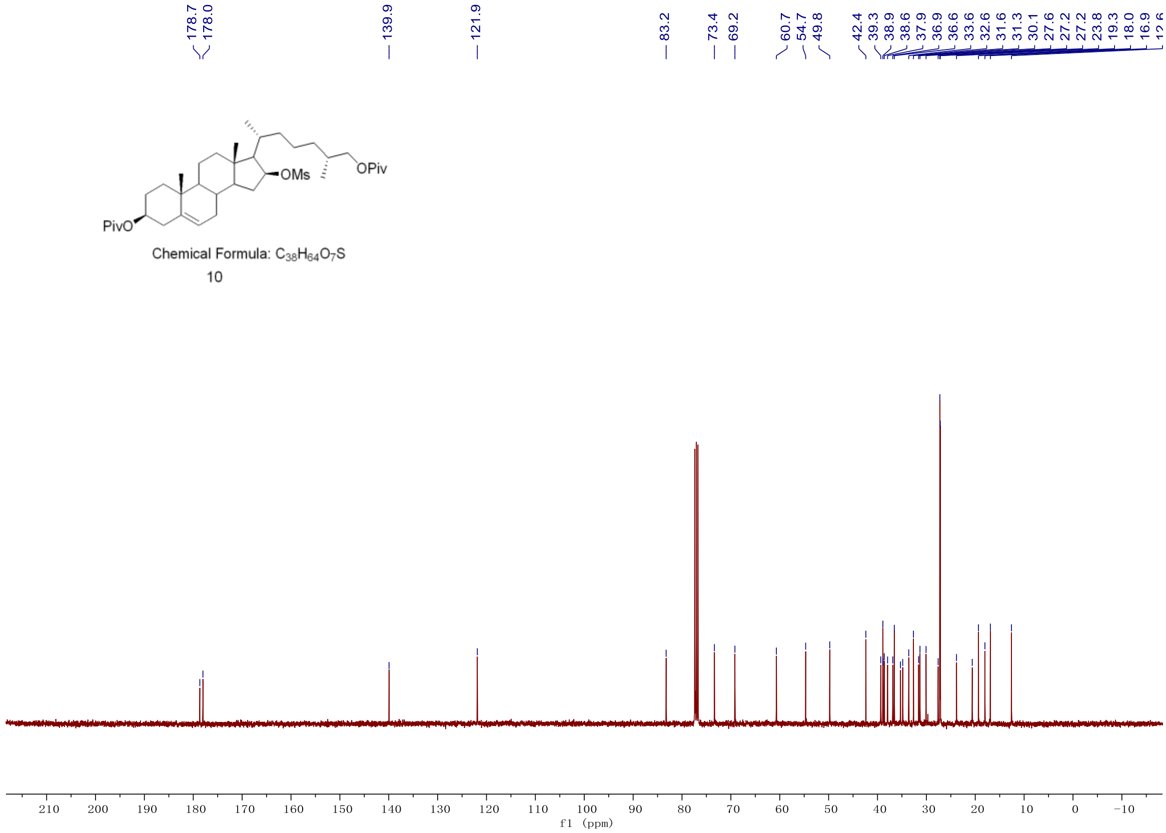


**$^{13}$C NMR spectrum (101 MHz, CDCl$_3$) of compound 10.**

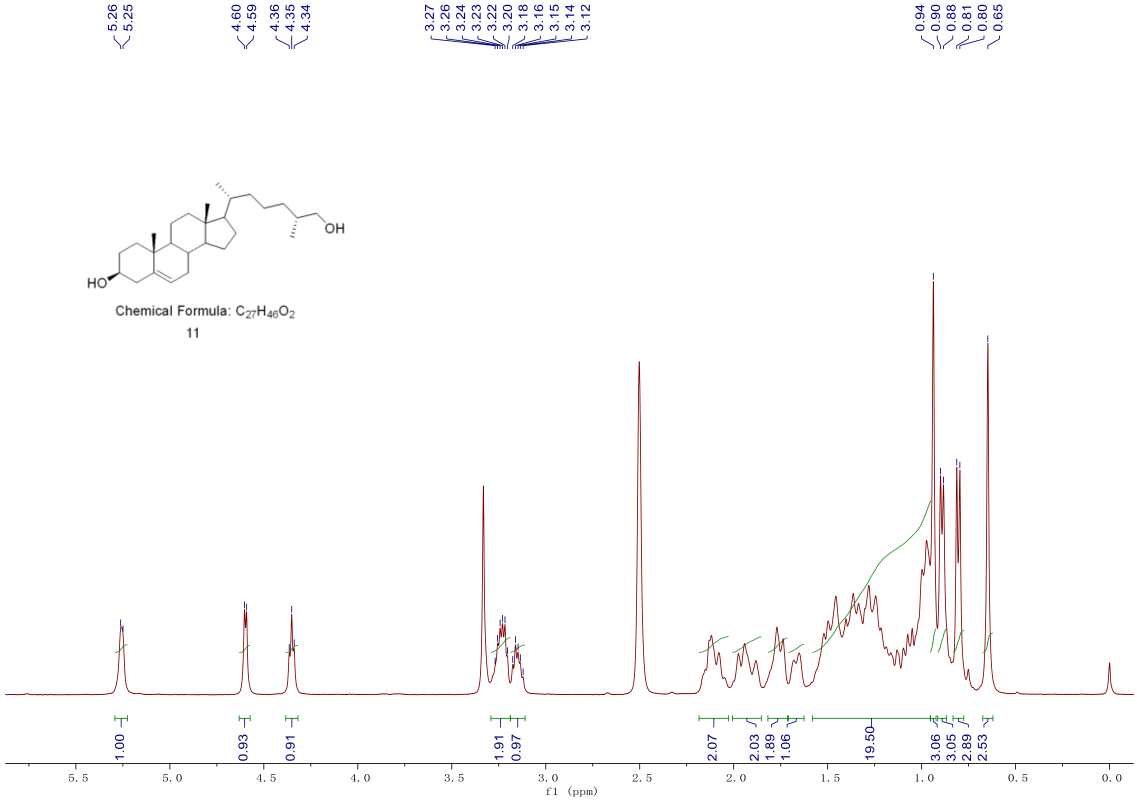


**$^{1}$H NMR spectrum (400 MHz, DMSO-d6) of compound 11.**

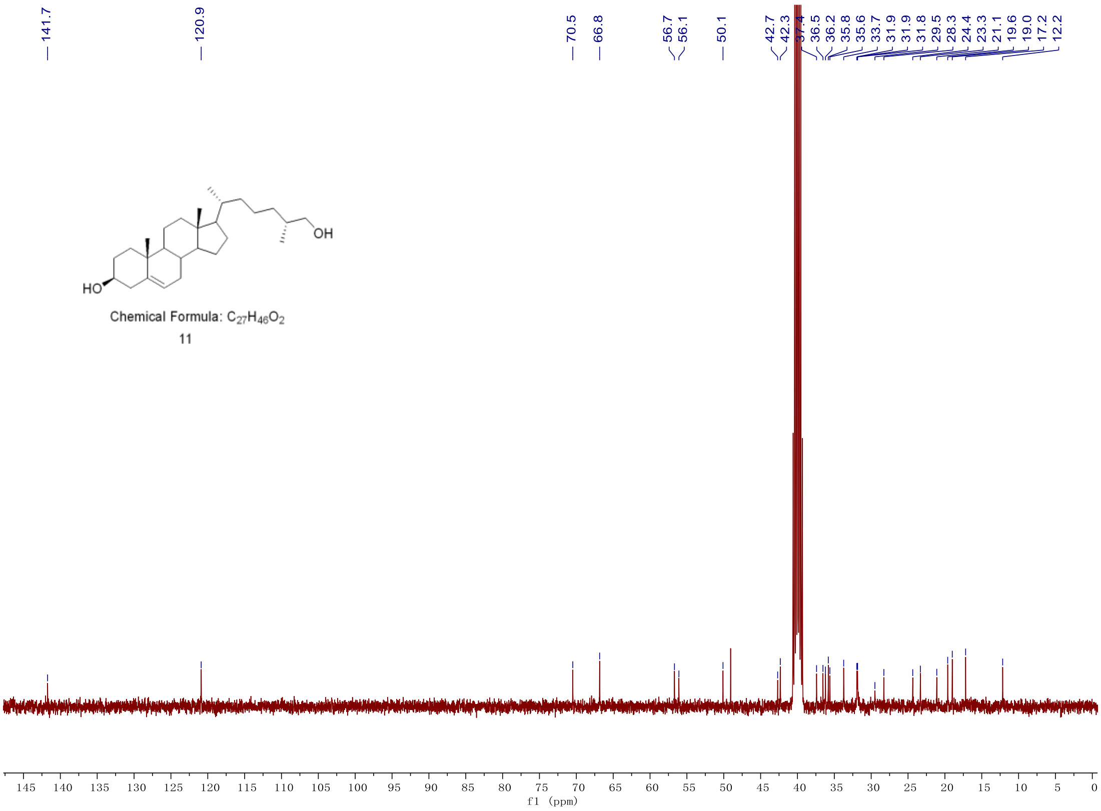


**$^{13}$C NMR spectrum (101 MHz, DMSO-d6) of compound 11.**

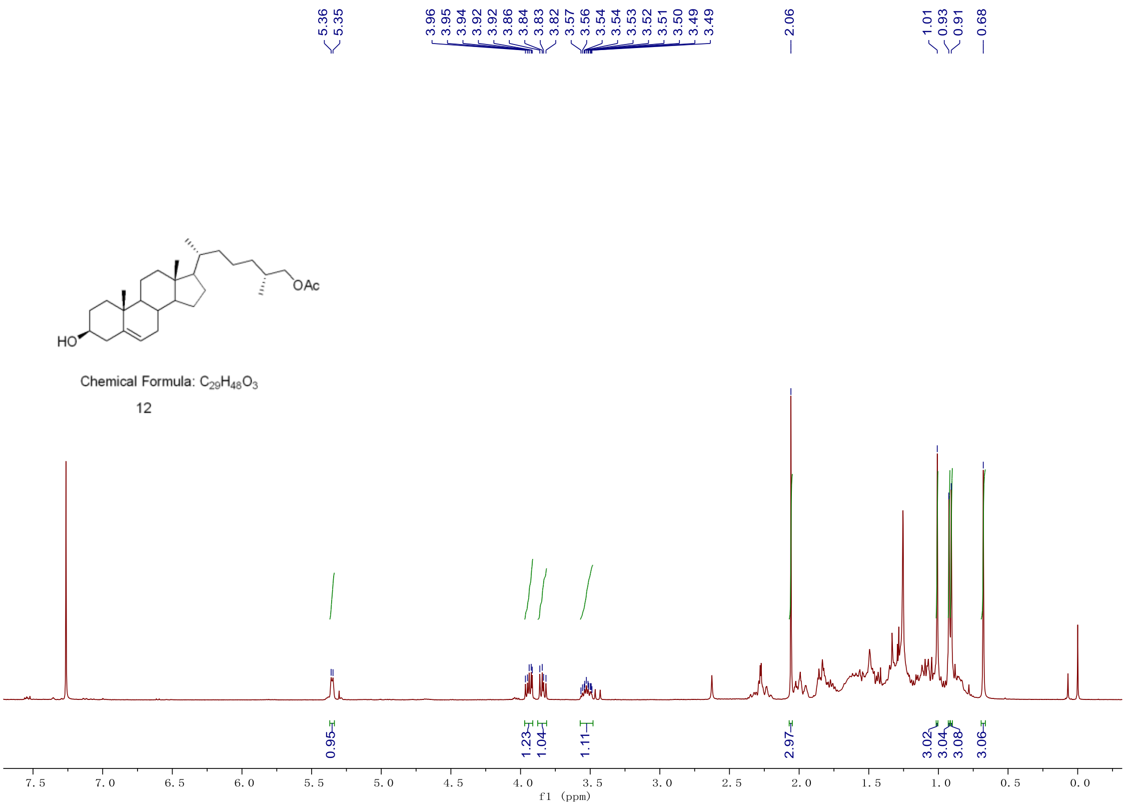


**$^{1}$H NMR spectrum (400 MHz, CDCl$_3$) of compound 12.**

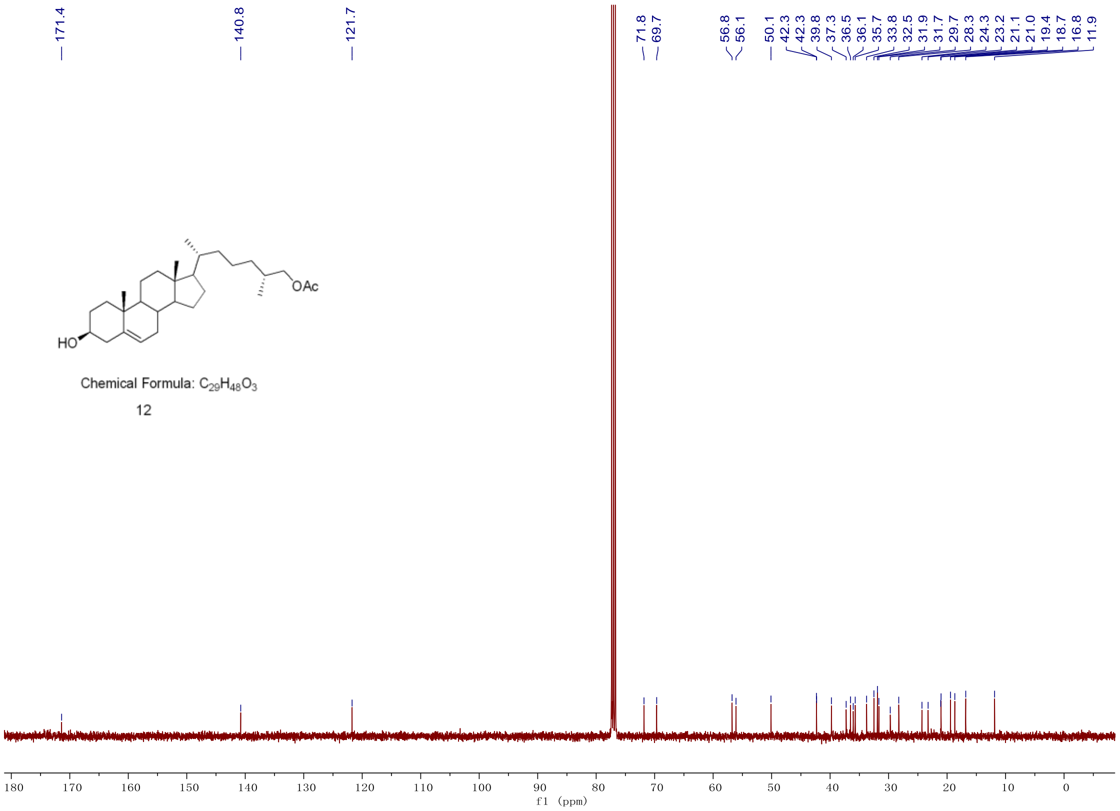


**$^{13}$C NMR spectrum (101 MHz, CDCl$_3$) of compound 12.**

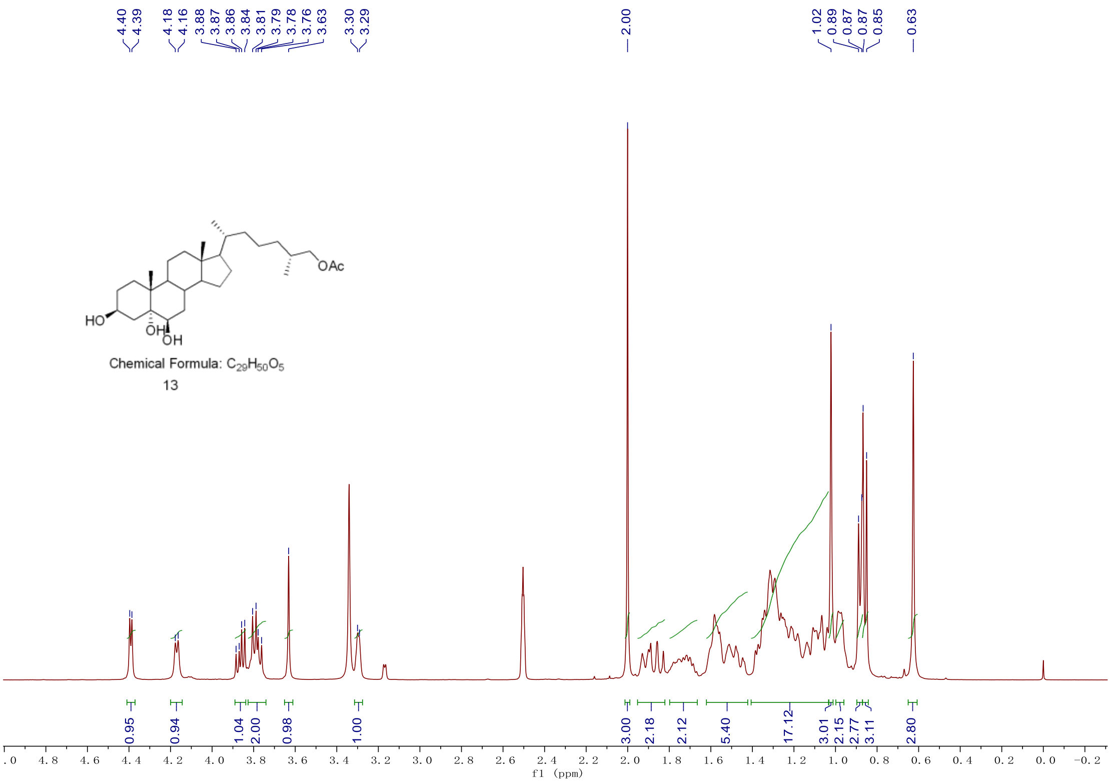

$^1H$ NMR spectrum (400 MHz, DMSO-$d_6$) of compound 13.

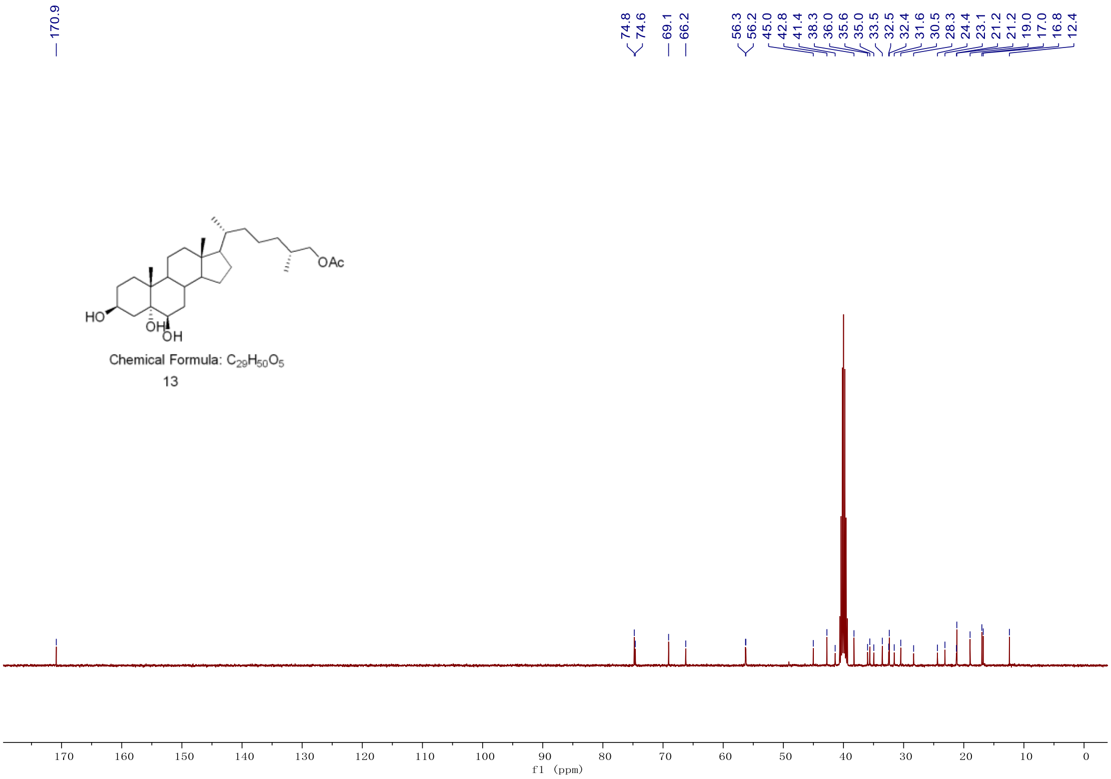

$^{13}C$ NMR spectrum (101 MHz, DMSO-$d_6$) of compound 13.

（A）

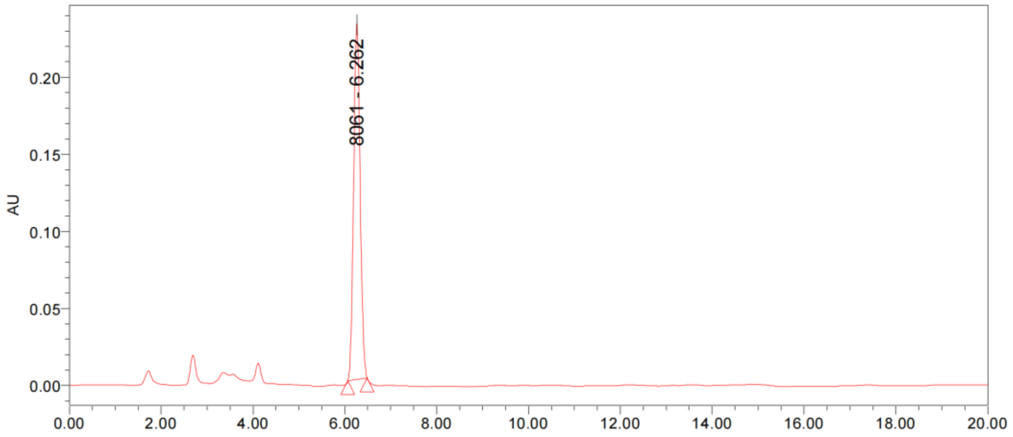


（B）

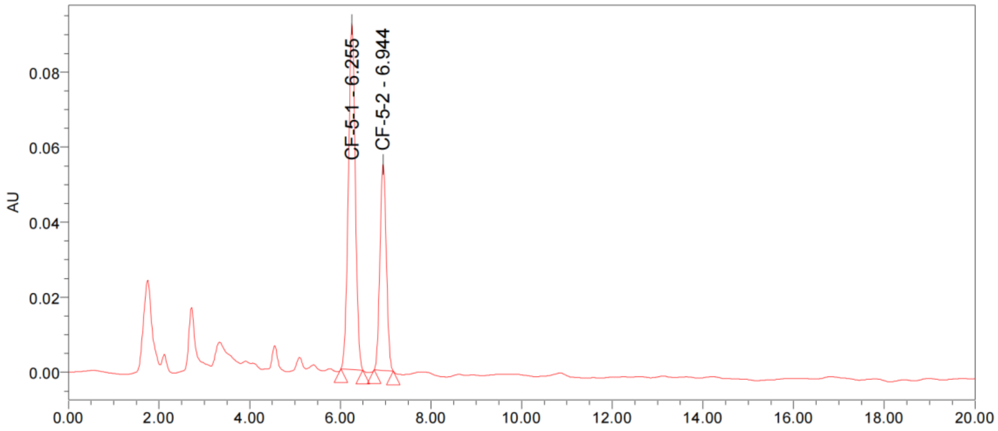


**Figure S6.** HPLC chromatograms of compounds (25*R*)-**5** and **5**. (A) HPLC chromatogram of compound (25R)-**5**. (B) HPLC chromatogram of compound **5**.

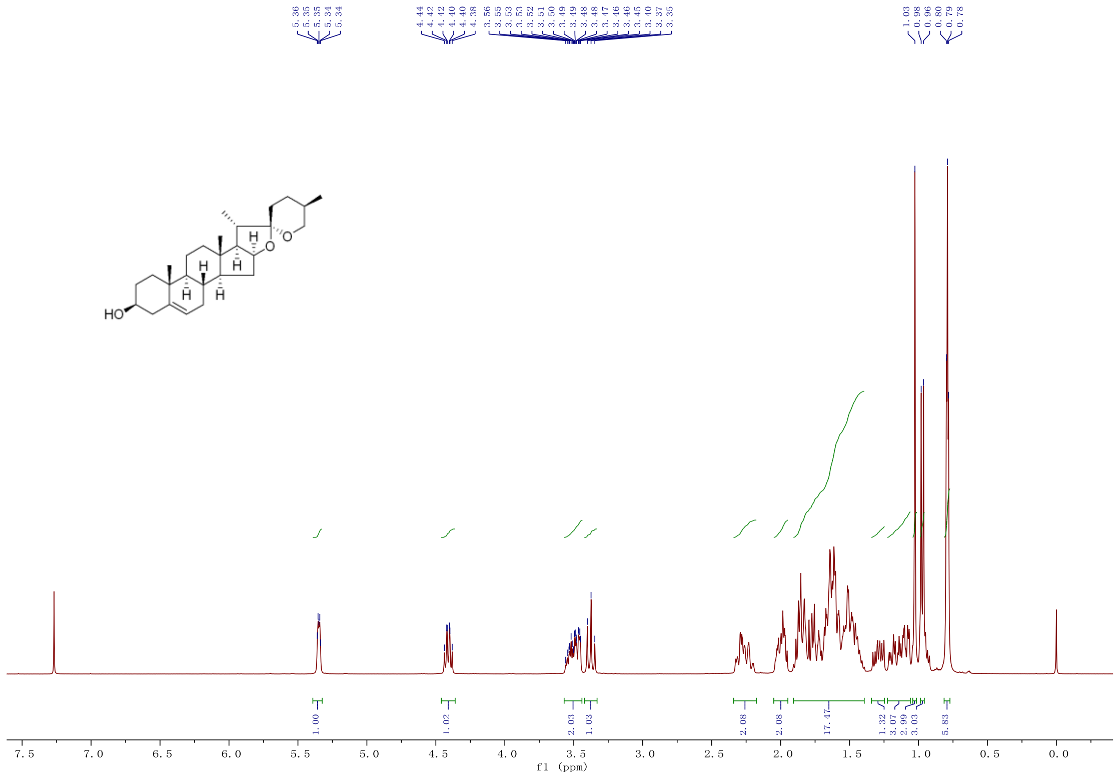

**¹H NMR spectrum (400 MHz, CDCl₃) of diosgenin.**
